# Relationship of blood heavy metals and osteoporosis among the middle-aged and elderly adults: A secondary analysis from NHANES 2013 to 2014 and 2017 to 2018

**DOI:** 10.3389/fpubh.2023.1045020

**Published:** 2023-03-14

**Authors:** Zengfa Huang, Xiang Wang, Hui Wang, Shutong Zhang, Xinyu Du, Hui Wei

**Affiliations:** ^1^Department of Radiology, The Central Hospital of Wuhan, Tongji Medical College, Huazhong University of Science and Technology, Wuhan, Hubei, China; ^2^Department of Radiology, The Central Hospital of Wuhan Base, Hubei University of Medicine, Shiyan, Hubei, China; ^3^Department of Orthopedics, The Affiliated Changzhou No. 2 People's Hospital of Nanjing Medical University, Changzhou, Jiangsu, China

**Keywords:** osteoporosis, heavy metals, NHANES, risk factors, middle-aged and older population

## Abstract

**Objective:**

This study aimed to assess the relationship between blood heavy metals and a higher prevalence of osteoporosis in middle-aged and elderly US adults using the National Health and Nutritional Examination Surveys (NHANES).

**Methods:**

The secondary data analysis was performed using the data of NHANES 2013–2014 and 2017–2018. We used the information, including physical examination, laboratory tests, questionnaires, and interviews, provided by participants in NHANES. Logistic regression and weighted quantile sum (WQS) regression models were used to explore the relationships between levels of blood heavy metals and a higher prevalence of osteoporosis.

**Results:**

A total of 1,777 middle-aged and elderly participants were analyzed in this study, comprising 115 participants with osteoporosis and 1,662 without osteoporosis. Adjusted model 1 showed a significant positive relationship between cadmium (Cd) levels and a higher prevalence of osteoporosis (quartile 2, OR = 7.62; 95% CI, 2.01–29.03; *p* = 0.003; quartile 3, OR = 12.38; 95% CI, 3.88–39.60; *p* < 0.001; and quartile 4, OR = 15.64; 95% CI, 3.22–76.08; *p* = 0.001). The fourth quartile of selenium (Se) level (OR = 0.34; 95% CI, 0.14–0.39; *p* < 0.001) led to a lower prevalence of osteoporosis and exerted a protective effect on model 1. Other models produced similar results to those of model 1. A subgroup analysis showed that Cd levels were positively related to a higher prevalence of osteoporosis in all three models in women, while this relationship was not found in men. The fourth quartile of the Se level was related to a lower prevalence of osteoporosis in both male and female analyses. A significant positive relationship was found between the blood Cd level and a higher prevalence of osteoporosis in the non-smoking subgroup. Blood Se level showed a protective effect on the fourth quartile in both the smoking and non-smoking subgroups.

**Conclusion:**

Blood Cd level aggravated the prevalence of osteoporosis, while blood Se level could be a protective factor in osteoporosis among the US middle-aged and older populations.

## Introduction

Osteoporosis is a systemic metabolic disease and remains a global health problem, which is increasingly becoming common in both developing and developed countries (1). It is estimated that there were 10.2 million cases of osteoporosis among the US population aged over 50 years in 2010 and that this number will reach 13.5 million by 2030 (2, 3). Osteoporosis is characterized by a loss of bone mineral density (BMD), which leads to an increased risk of fragility fractures and thus an increased economic and medical burden on the patient (4). As the definition of osteoporosis either by experts or by an explanation based on histology did not prove to be practical for patient care, a panel of the World Health Organization (WHO) defined osteoporosis as BMD values of 2.5 standard deviations (SDs) or more below the mean of the young adult reference group (5). There are many risk factors for osteoporosis and BMD reduction, including older age and female gender (6). In recent years, it has been hypothesized that heavy metals may be associated with the risk of degenerative diseases and fractures (7).

Heavy metals have been demonstrated to be associated with adverse health effects. Moreover, exposure to heavy metals in the environment will affect genes and increase disease susceptibility (8). The accumulation of heavy metals in the human body will change hormone metabolism and lead to vasoconstriction, thus leading to adult diseases (7). Accordingly, a recent study revealed that the accumulation of blood heavy metals in bones increases bone resorption and changes bone mineral content, which will eventually lead to osteoporosis and bone fracture (9). Several studies indicated a negative correlation between daily or long-time exposure to cadmium (Cd), lead (Pb), mercury (Hg), and BMD (10). However, no significant correlation between dietary intake of these heavy metals and bone parameters was observed (11).

The relationship between blood heavy metals and the risk of osteoporosis has only been reported in observational studies involving small sample sizes (12). Moreover, several bodies of research determined the exposure to heavy metals based on the urinary or environmental levels of heavy metals (13–15). Nevertheless, it is unclear whether blood heavy metal levels are associated with osteoporosis in the general aging population. Therefore, this study aimed to assess the relationship of blood heavy metals with a higher prevalence of osteoporosis in a US population of middle-aged and elderly people using the National Health and Nutritional Examination Surveys (NHANES). Investigation of the correlation between blood heavy metals and osteoporosis is important as people may experience cumulative exposure in some circumstances and osteoporosis is a threat to the aging population. An analysis of the relationship between aging and osteoporosis could help prevent osteoporosis and reduce the exposure of the aging population to the risk factors.

## Methods

### Study subjects

**Figure 1:**
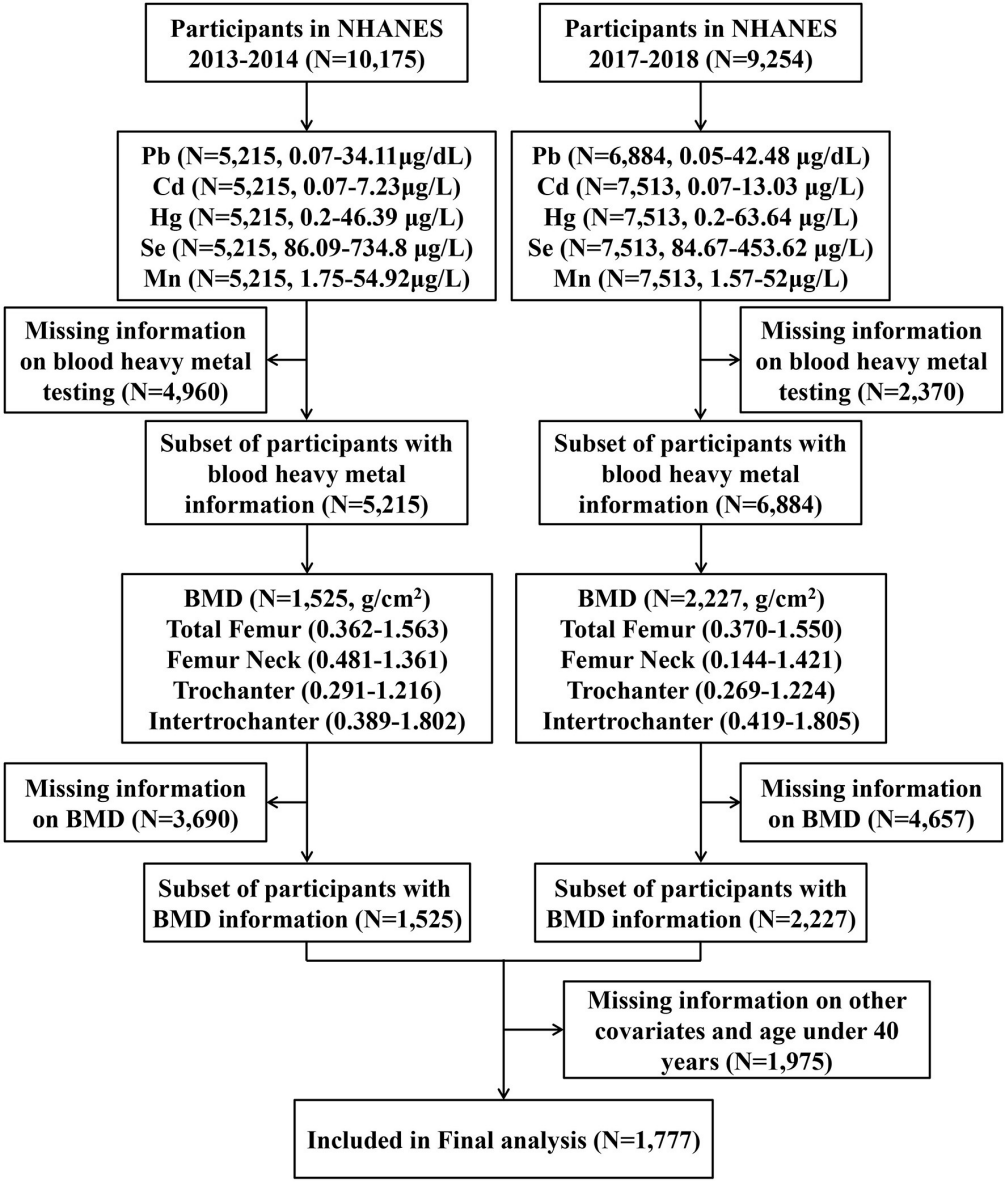
Flowchart of the study population selection.

This study was performed as a secondary analysis using the data collected in NHANES 2013–2014 and 2017–2018, and the data were collected by physical examination, laboratory tests, questionnaires, and interviews. Details of the NHANES can be found on the website of the American Centers for Disease Control and Prevention (https://www.cdc.gov/nchs/nhanes). In the present study, we first enrolled all participants from NHANES 2013 to 2014 (*N* = 10,175) and 2017 to 2018 (N = 9,254). Then, we excluded participants with incomplete information on blood heavy metals (*N* = 7,330), BMD data (*N* = 8,347), and missing basic information as well as those aged below 40 years (*N* = 1,975). In the end, a total of 1,777 individuals were included in the final analysis (Figure 1).

### Evaluation of osteoporosis

The BMD values at different sites (the total femur, the femur neck, the trochanter, and the trochanter intertrochanter) were measured using dual-energy x-ray absorptiometry (DXA) with Hologic QDR-4500A fan-beam bone densitometers (Hologic, Inc., Bedford, MA, USA). The regions of the proximal femur of the left hip were routinely examined. An examination of the left hip was replaced by the right hip on the condition that the participant reported having replacement or metal objects in the left leg. Any participant who was pregnant, who weighed over 300 pounds, or had a history of radiographic contrast material, fractures, replacements, or pins in both hips was excluded from the DXA examination.

Osteoporosis was defined as BMD values of 2.5 standard deviations (SDs) or more below the mean of the young adult reference group according to the guidelines of the World Health Organization (WHO) (5). This study assessed osteoporosis in four regions of the femur: the total femur, the femur neck, the trochanter, and the intertrochanter, and the thresholds were 0.67 g/cm^2^, 0.56 g/cm^2^, 0.46 g/cm^2^, and 0.79 g/cm^2^, respectively (16). Osteoporosis in any femoral region was defined as overall osteoporosis.

### Assessment of heavy metals

After performing the step involving a simple dilution sample preparation, blood heavy metals, such as Pb, Cd, total Hg, selenium (Se), and manganese (Mn), were directly measured in whole blood samples by mass spectrometry. To carry out a uniform distribution of cellular components, a small amount of whole blood was extracted from a larger sample of whole blood after mixing during the dilution phase. Dilution of blood includes simple dilution of 1 part sample + 1 part water + 48 parts diluent during sample preparation before analysis. Liquid samples were introduced into the mass spectrometer through the inductively coupled plasma ionization ion source (17).

### Ascertainment of covariates

Information on demography and lifestyle factors was collected by trained personnel, according to the statement mentioned on the NHANES website. Demographic characteristics included age (years), gender (male or female), race/ethnicity (Mexican, non-Hispanic white, non-Hispanic black, Mexican American, and other races), educational level (less than 9th grade, 9–11th grades, high school, some college, or college graduate), and physical activity (Yes and No). Alcohol consumption was defined as < 12 or ≥12 alcoholic drinks per year. Smoking status was categorized into never smokers or current smokers. Exposure to secondhand smoke was indicated as no one in the household is a smoker or ≥1 one member in the household is a smoker. Sedentary behavior was defined as sitting for more than 6 h a day, which does not include time spent sleeping. The medical examinations were carried out in mobile centers. Body mass index (BMI, kg/m^2^) was classified as < 25, 25–30, or >30. Diabetes was defined as reporting a previous diagnosis or reaching a fasting glucose level of ≥126 mg/dl. Hypertension status was defined as reporting a previous diagnosis (yes or no). Arthritis was defined as a doctor ever diagnosing one to have had arthritis. A thyroid problem was defined as a doctor ever diagnosing one to have had a thyroid problem. Hypercholesterolemia was defined as total cholesterol values ≥240 mg/dl. The estimated glomerular filtration rate (GFR) was calculated based on age, gender, and serum creatinine according to the Chronic Kidney Disease Epidemiology Collaboration equation (18). The annual household income was classified as < $20,000, $20,000–$34,999, $34,999–$74,999, or ≥$75,000.

### Statistical analysis

All analyses considered complex survey design factors, including sample weights, clustering, and stratification, with instructions for using NHANES data. Four-year sampling weights were calculated by multiplying the sampling weights provided by NHANES for 2-year cycles by two. Data were expressed as mean ± standard derivation (SD) for continuous variables and numbers (percentages) for categorical variables. We used Student's *t*-test for continuous variables and the Chi-square test for categorical variables. As the blood heavy metals displayed non-normal distribution, categorical groups rather than continuous values were used in statistical analysis. The levels of blood heavy metals (Pb, Cd, Hg, Mn, and Se) were categorized into one of the four groups based on quartiles (quartile 1: < 25th percentile, quartile 2: 25th−50th percentile, quartile 3: 50th−75th percentile, and quartile 4: >75th percentile). Categorical groups and continuous analysis of blood heavy metals of logistic regressions with weights were used to estimate the odds ratio (ORs) with 95% confidence intervals (95% CIs) for the relationships between blood heavy metals and the prevalence of osteoporosis. Model 1 was adjusted for gender, age, and race, and model 2 was further adjusted for all the covariates. Blood heavy metals were evaluated by using quartile 1 as the reference. We further used weighted quantile sum regression (WQS) models with positive and negative directionality modes for the mixed effects. A two-sided value of *P* of < 0.05 was considered statistically significant. All the analyses were conducted using SAS version 9.4 (SAS Institute Inc., Cary, NC, USA) and R software version 4.2.2 (Vienna, Austria).

## Results

### Characteristics of participants

The present study included a total of 1,777 participants, involving 115 of them with osteoporosis and 1,662 without osteoporosis. The weighted average age was 58.9 ± 0.4 years and 50.4% of them were men. Table 1 shows the basic characteristics of the study participants. Compared with the non-osteoporosis group, participants with osteoporosis were older and were more likely to be women. These participants also had a higher prevalence of normal BMI, arthritis, thyroid problems, a lower GFR, and a lower annual household income. There were no significant differences in race, education, smoking status, exposure to secondhand smoke, alcohol consumption, physical activity, sedentary behavior, hypercholesterolemia, or history of diabetes or hypertension between the osteoporosis group and the non-osteoporosis group (Table 1). Characteristics of participants based on the levels of heavy metals in their blood are listed in Supplementary Tables S1–S5. The weighted geometric mean (GM) and quartiles of concentrations of blood heavy metals are listed in Table 2.

**Table 1:** Characteristics of participants with and without osteoporosis in the enrolled population of NHANES.

**Characteristic**	**Total (*N* = 1,777)**	**No osteoporosis (*N* = 1,662)**	**Osteoporosis (*N* = 115)**	***T/χ^2^***	***P*-value**
Age (weighted years, mean ± SD)	58.9 ± 0.4	58.4 ± 0.4	66.9 ± 1.5	32.75	< 0.001
Gender, no. (weighted %)^a^				28.24	< 0.001
Men	903 (50.4)	865 (52.0)	38 (25.2)		
Women	874 (49.6)	797 (48.0)	77 (74.8)		
Race, no. (weighted %)^a^				7.54	0.342
Mexican	218 (8.8)	209 (9.2)	9 (3.5)		
Other Hispanic	165 (6.0)	154 (5.8)	11 (9.7)		
Non-Hispanic white	788 (61.8)	728 (62.0)	60 (59.3)		
Non-Hispanic Black	351 (13.4)	333 (13.3)	18 (14.0)		
Other Race	255 (10.0)	238 (9.7)	17 (13.5)		
Education, no. (weighted %)^a^				7.53	0.217
Less than 9th grade	209 (9.5)	187 (9.3)	22 (12.3)		
9–11 grade	503 (25.1)	470 (24.7)	33 (31.5)		
High school graduate or equivalent	423 (24.0)	398 (23.8)	25 (27.2)		
Some college or AA degree	304 (17.5)	288 (17.8)	16 (13.5)		
College graduate or above	338 (23.9)	319 (24.4)	19 (15.5)		
BMI, no. (weighted %)^a^				34.40	< 0.001
Normal (< 25)	442 (23.5)	387 (22.0)	55 (46.0)		
Overweight (25–30)	662 (38.3)	622 (38.6)	40 (33.8)		
Obesity (>30)	673 (38.2)	653 (39.4)	20 (20.2)		
Smoking status, no. (weighted %)^a^				1.25	0.350
Yes	382 (18.9)	354 (18.7)	28 (23.1)		
No	1,395 (81.1)	1,308 (81.3)	87 (76.9)		
Physical activity, no. (weighted %)^a^				1.72	0.277
Yes	350 (20.8)	332 (21.1)	18 (23.1)		
No	1,427 (79.2)	1,330 (78.9)	97 (76.9)		
Diabetes, no. (weighted %)^a^				1.03	0.406
Yes	362 (16.9)	345 (17.1)	17 (15.8)		
No	1,415 (83.1)	1,317 (82.9)	98 (84.2)		
Hypertension, no. (weighted %)^a^				3.59	0.162
Yes	878 (45.0)	814 (44.5)	64 (53.9)		
No	899 (55.0)	848 (55.5)	51 (46.1)		
Alcohol consumption, no. (weighted %)^a^				0.02	0.871
≥12 times per year	247 (13.8)	234 (13.8)	13 (14.3)		
< 12 times per year	1,530 (86.2)	1,428 (86.2)	102 (85.7)		
Exposure to secondhand smoke, no. (weighted %)^a^				1.77	0.441
Yes	452 (22.5)	421 (22.2)	31 (27.8)		
No	1,325 (77.5)	1,241 (77.8)	84 (72.2)		
Sedentary behavior, no. (weighted %)^a^				0.05	0.872
Yes	1,025 (60.3)	957 (60.3)	68 (59.3)		
No	752 (39.7)	705 (39.7)	47 (40.7)		
Arthritis, no. (weighted %)^a^				8.71	0.016
Yes	677 (39.4)	617 (38.5)	60 (53.1)		
No	1,100 (60.6)	1,045 (61.5)	55 (46.9)		
Thyroid problems, no. (weighted %)^a^				5.54	0.038
Yes	253 (16.4)	436 (15.9)	32 (24.7)		
No	1,774 (83.6)	1,653 (84.1)	121 (75.3)		
Hypercholesterolemia, no. (weighted %)^a^				1.15	0.301
Yes	763 (44.4)	712 (44.7)	51 (39.3)		
No	1,014 (55.6)	950 (55.3)	64 (60.7)		
GFR, no. (weighted %)^a^				15.88	< 0.001
< 60 ml/min/1.73 m^2^	265 (13.2)	235 (12.5)	30 (25.7)		
60–90 ml/min/1.73 m^2^	810 (47.7)	762 (47.9)	48 (44.4)		
≥90 ml/min/1.73 m^2^	702 (39.1)	665 (39.7)	37 (29.8)		
Annual household income, no. (weighted %)^a^				26.77	< 0.001
$0–$19,999	327 (11.2)	299 (10.8)	28 (18.5)		
$20,000–$34,999	332 (13.8)	303 (13.3)	29 (20.6)		
$35,000–$74,999	421 (24.3)	390 (23.7)	31 (34.5)		
$75,000 and over	697 (50.7)	670 (52.2)	27 (26.4)		

^a^Numbers of participants are unweighted. All percentage estimates are weighted.

**Table 2:** Blood levels of heavy metals by osteoporosis status in US middle-aged and elderly people from NHANES^a^.

**Heavy metals**	**No osteoporosis (*****N*** = **1,162)**					**Osteoporosis (*****N*** = **115)**				
	**GM**	**Q1**	**Q2**	**Q3**	**Q4**	**GM**	**Q1**	**Q2**	**Q3**	**Q4**
Lead (μg/dL)	1.16	< 0.77	0.78–1.13	1.13–1.64	>1.64	1.21	< 0.82	0.82–1.23	1.23–1.83	>1.83
Cadmium (μg/L)	0.32	< 0.18	0.18–0.28	0.28–0.51	>0.51	0.55	< 0.30	0.30–0.43	0.43–0.84	>0.84
Mercury (μg/L)	0.98	< 0.47	0.47–0.92	0.92–1.87	>1.87	0.86	< 0.43	0.43–0.75	0.75–1.46	>1.46
Selenium (μg/L)	196.91	< 181.41	181.41–196.81	196.81–211.28	>211.28	182.31	< 165.95	165.95–183.39	183.39–198.8	>198.8
Manganese (μg/L)	8.90	< 7.22	7.22–8.84	8.84–10.64	>10.64	8.95	< 7.0	7.0–8.87	8.87–11.45	>11.45

^a^All GM and Q1–Q4 estimates are weighted.

### Relationships of blood heavy metals with osteoporosis

Tables 3, 4 show the relationships between levels of blood heavy metals and osteoporosis using univariate logistic regression and multivariate logistic regression, respectively. Cd levels had a positive relationship with osteoporosis. Moreover, there is a negative relationship between Se levels and osteoporosis. The adjusted model 1 (adjusted by age, gender, and race) showed a significant positive relationship between Cd levels and osteoporosis (quartile 2, OR = 7.62; 95% CI, 2.01–29.03; *p* = 0.003; quartile 3, OR = 12.38; 95% CI, 3.88–39.60; *p* < 0.001; and quartile 4, OR = 15.64; 95% CI, 3.22–76.08; *p* = 0.001). In Se element analysis, taking the first quartile as a reference, the fourth quartile (OR = 0.34; 95% CI, 0.14–0.39; *p* < 0.001) led to a lower prevalence of osteoporosis and exerted a protective effect on model 1. However, the second (OR = 0.52; 95% CI, 0.27–1.02; *p* = 0.056) and third quartiles (OR = 0.46; 95% CI, 0.21–1.03; *p* = 0.059) were associated with a numerically decreased prevalence of osteoporosis with borderline significance. The third quartile of the Mn level showed a borderline negative significant relationship with osteoporosis (OR = 0.47; 95% CI, 0.22–0.99; *p* = 0.049) in model 1. Pb and Hg have no relationship with osteoporosis. Model 2 (adjusted by age, gender, race, education, BMI, arthritis, thyroid problems, GFR, and annual household income) and model 3 (adjusted by age, gender, race, education, smoke, diabetes, hypertension, physical activity, BMI, alcohol consumption, exposure to secondhand smoke, sedentary behavior, arthritis, thyroid problems, hypercholesterolemia, GFR, and annual household income) produced similar results to those of model 1 for the relationship between blood heavy metals and the prevalence of osteoporosis.

**Table 3:** Univariate logistic analysis of osteoporosis in NHANES.

**Characteristic**	**Univariate**		
	**OR**	**95% CI**	**P-value**
Age (≥60 years)	3.38	1.74–6.56	< 0.001
Male	0.21	0.20–0.48	< 0.001
**Race**			
Mexican	0.28	0.08–0.96	0.043
Other Hispanic	1.21	0.42–3.46	0.723
Non-Hispanic white	0.69	0.28–1.68	0.416
Non-Hispanic Black	0.76	0.26–2.25	0.619
**Education**			
Less than 9th grade	2.08	1.03–4.20	0.042
9–11 grade	2.01	0.97–4.18	0.062
High school graduate or equivalent	1.80	0.80–4.05	0.157
Some college or AA degree	1.20	0.56–2.57	0.644
**BMI**			
Overweight (25–30)	0.25	0.14–0.44	< 0.001
Obesity (>30)	0.42	0.27–0.66	< 0.001
Smoking status	1.31	0.75–2.29	0.348
Physical activity	0.70	0.36–1.35	0.284
Diabetes	0.74	0.37–1.50	0.405
Hypertension	1.46	0.85–2.52	0.170
Alcohol consumption	1.04	0.63–1.73	0.871
Exposure to secondhand smoke	1.35	0.63–2.88	0.441
Sedentary behavior	0.96	0.56–1.63	0.872
Arthritis	1.80	1.1–2.94	0.018
Thyroid problems	1.73	1.02–2.93	0.041
Hypercholesterolemia	0.80	0.53–1.22	0.305
**GFR**			
< 60 ml/min/1.73 m^2^	2.75	1.57–4.81	< 0.001
60–90 ml/min/1.73 m^2^	1.24	0.78–1.95	0.367
**Annual household income**			
$0–$19,999	3.40	1.53–7.51	0.003
$20,000–$34,999	3.06	1.28–7.32	0.012
$35,000–$74,999	2.88	1.40–5.90	0.004
**Pb**			
Q2	0.95	0.43–2.08	0.892
Q3	1.19	0.63–2.27	0.531
Q4	1.33	0.69–2.56	0.399
**Cd**			
Q2	8.58	2.68–27.40	< 0.001
Q3	15.19	5.95–38.82	< 0.001
Q4	17.90	4.70–68.20	< 0.001
**Hg**			
Q2	0.79	0.46–1.38	0.113
Q3	0.96	0.57–1.62	0.875
Q4	0.53	0.24–1.66	0.413
**Se**			
Q2	0.51	0.28–0.93	0.027
Q3	0.36	0.19–0.70	0.002
Q4	0.22	0.13–0.35	< 0.001
**Mn**			
Q2	0.78	0.38–1.62	0.505
Q3	0.69	0.37–1.31	0.256
Q4	1.24	0.69–2.23	0.478

All OR (95% CI) estimates are weighted.

**Table 4:** Multivariate logistic analysis of osteoporosis in NHANES.

**Characteristic**	**Model 1**			**Model 2**			**Model 3**		
	**OR**	**95% CI**	**P-value**	**OR**	**95% CI**	**P-value**	**OR**	**95% CI**	**P-value**
Age (≥60 years)	3.78	1.96–7.28	< 0.001	3.58	1.81–7.09	< 0.001	3.39	1.63–7.05	0.001
Male	0.37	0.24–0.59	< 0.001	0.38	0.23–0.61	< 0.001	0.38	0.23–0.63	< 0.001
**Race**									
Mexican	0.28	0.09–0.83	0.021	0.26	0.09–0. 74	0.012	0.25	0.08–0.76	0.014
Other Hispanic	1.09	0.39–3.10	0.867	1.01	0.36–2.82	0.982	0.97	0.35–2.66	0.954
Non-Hispanic white	0.64	0.28–1.65	0.353	0.69	0.25–1.90	0.468	0.65	0.23–1.81	0.412
Non-Hispanic Black	0.64	0.21–1.96	0.434	0.69	0.21–2.22	0.530	0.70	0.22–2.28	0.559
**Education**									
Less than 9th grade				1.60	0.60–4.33	0.351	2.21	0.78–6.22	0.137
9–11 grade				1.50	0.57–3.93	0.411	1.90	0.64–5.65	0.247
High school graduate or equivalent				1.52	0.56–4.16	0.416	1.70	0.58–5.04	0.336
Some college or AA degree				1.27	0.48–3.37	0.637	0.44	1.56–1.27	0.543
**BMI**									
Overweight (25–30)				0.43	0.26–0.72	0.002	0.41	0.26–0.66	< 0.001
Obesity (>30)				0.23	0.12–0.44	< 0.001	0.21	0.10–0.43	< 0.001
Smoking status							0.44	0.16–1.27	0.130
Physical activity							0.84	0.41–1.72	0.629
Diabetes							0.73	0.31–1.69	0.456
Hypertension							1.15	0.65–2.05	0.625
Alcohol consumption							0.62	0.34–1.13	0.115
Exposure to secondhand smoke							1.26	0.50–3.18	0.637
Sedentary behavior							1.18	0.58–2.41	0.648
Arthritis				1.32	0.72–2.41	0.367	1.37	0.75–2.53	0.310
Thyroid problems				1.05	0.58–1.88	0.881	1.04	0.58–1.89	0.892
Hypercholesterolemia							0.92	0.55–1.52	0.731
**GFR**									
< 60 ml/min/1.73 m^2^				1.44	0.75–2.76	0.272	1.37	0.73–2.58	0.329
60–90 ml/min/1.73 m^2^				0.79	0.48–1.31	0.361	0.79	0.48–1.32	0.374
**Annual household income**									
$0–$19,999				1.96	0.83–4.61	0.124	1.88	0.77–4.54	0.164
$20,000–$34,999				2.68	1.12–6.37	0.026	2.46	0.97–6.20	0.057
$35,000–$74,999				2.86	1.28–6.39	0.010	2.84	1.25–6.48	0.013
**Pb**									
Q2	0.86	0.40–1.88	0.705	0.73	0.32–1.64	0.444	0.69	0.31–1.55	0.365
Q3	0.78	0.40–1.51	0.459	0.60	0.33–1.63	0.112	0.64	0.33–1.25	0.189
Q4	0.76	0.41–1.39	0.382	0.52	0.30–0.94	0.030	0.53	0.28–0.98	0.044
**Cd**									
Q2	7.62	2.01–29.03	0.003	10.01	2.16–46.39	0.003	10.01	2.05–48.83	0.004
Q3	12.38	3.88–39.60	< 0.001	14.23	3.59–56.39	< 0.001	14.15	3.31–60.52	< 0.001
Q4	15.64	3.22–76.08	0.001	13.51	2.22–82.29	0.004	17.98	2.54–127.07	0.004
**Hg**									
Q2	0.93	0.52–1.66	0.801	0.89	0.48–1.68	0.728	0.89	0.47–1.67	0.710
Q3	1.16	0.68–1.96	0.592	1.21	0.69–2.12	0.516	1.23	0.69–2.21	0.486
Q4	0.62	0.28–1.37	0.235	0.69	0.31–1.54	0.366	0.61	0.27–1.37	0.229
**Se**									
Q2	0.52	0.27–1.02	0.056	0.50	0.26–0.95	0.035	0.51	0.27–0.95	0.032
Q3	0.46	0.21–1.03	0.059	0.57	0.26–1.26	0.164	0.56	0.24–1.27	0.165
Q4	0.34	0.14–0.39	< 0.001	0.26	0.15–0.25	< 0.001	0.26	0.15–0.45	< 0.001
**Mn**									
Q2	0.53	0.21–1.30	0.163	0.61	0.28–1.35	0.225	0.62	0.29–1.31	0.212
Q3	0.47	0.22–0.99	0.049	0.57	0.25–1.29	0.177	0.53	0.23–1.22	0.135
Q4	0.71	0.33–1.54	0.390	0.97	0.49–1.93	0.940	0.90	0.46–1.79	0.770

Model 1: Adjusted by age, gender, and race.

Model 2: Adjusted by age, gender, race, education, BMI, arthritis, thyroid problems, GFR, and annual household income.

Model 3: Adjusted by age, gender, race, education, smoke, diabetes, hypertension, physical activity, BMI, alcohol consumption, exposure to secondhand smoke, sedentary behavior, arthritis, thyroid problems, hypercholesterolemia, GFR, and annual household income.

All OR (95% CI) estimates are weighted.

**Figure 2:**
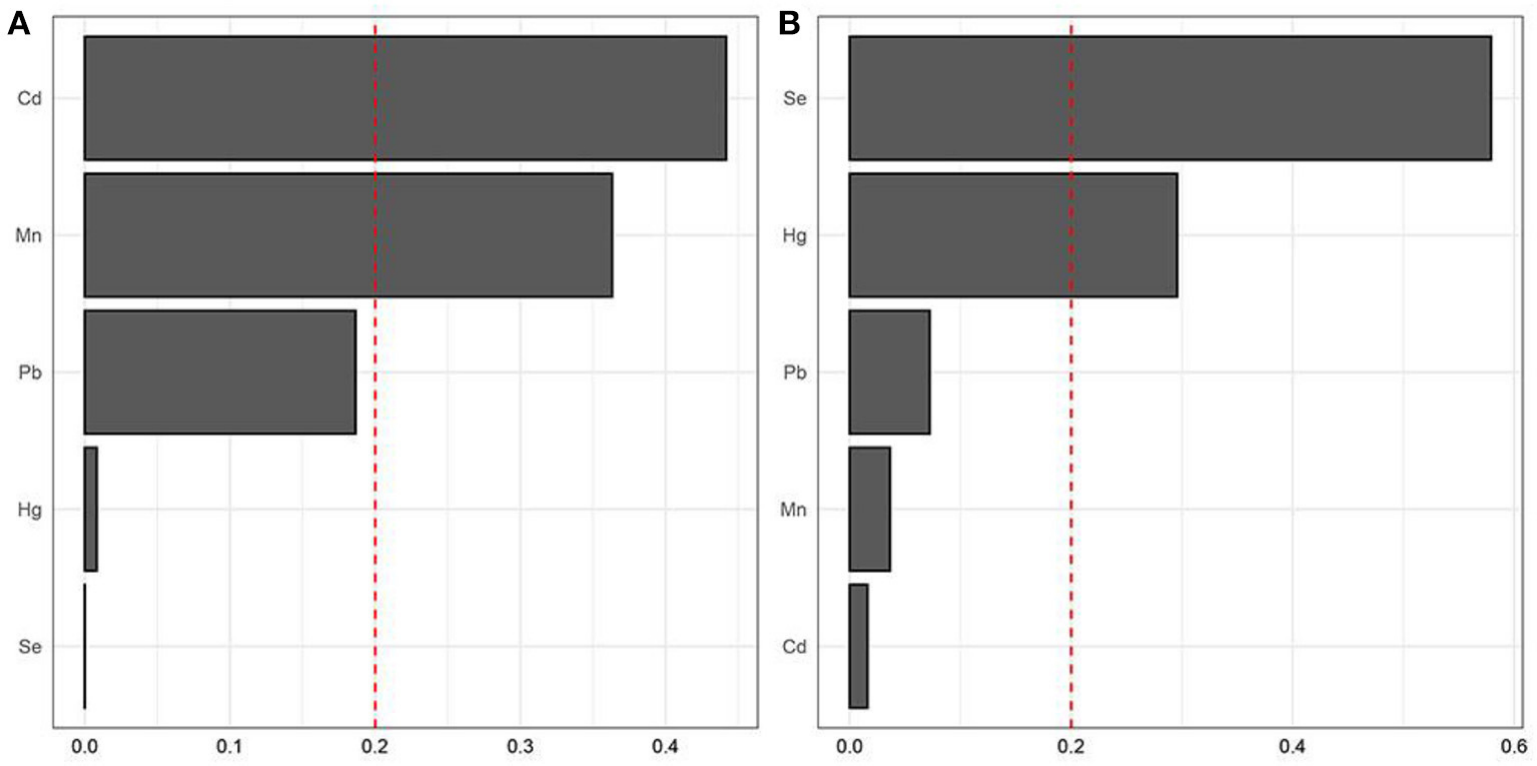
Identification of blood heavy metals in the mixture using the WQS model. **(A)** positive WQS model; **(B)** negative WQS model.

Blood heavy metals that make a major contribution to the whole relationship of the mixture (Pb, Cd, Hg, Se and Mn) were analyzed using the WQS models. The ranking of blood heavy metals was based on the probability of the maximum weight of blood heavy metals in the mixture. WQS with a positive directional mode showed that Cd was positively related to a higher prevalence of osteoporosis, while Se was negatively related to the higher prevalence of osteoporosis (Figure 2).

### Subgroup analysis

In the subgroup analysis stratified by gender, logistic regression analysis revealed that the third and fourth quartiles of the Se level were associated with a lower prevalence of osteoporosis (OR = 0.31; 95% CI, 0.10–0.92; *p* = 0.035, OR = 0.16; 95% CI, 0.04–0.63; *p* = 0.009, respectively) in model 3 in men. There was no relationship between Cd levels and the prevalence of osteoporosis in men (Supplementary Table S6). However, in women, Cd levels were shown to be positively related to a higher prevalence of osteoporosis (quartile 2, OR = 14.11; 95% CI, 2.12–94.13; *p* = 0.006; quartile 3, OR = 30.55; 95% CI, 5.90–158.11; *p* < 0.001; and quartile 4, OR = 27.00; 95% CI, 3.34–218.29; *p* = 0.002) in model 3. The fourth quartile of the Se level was related to a lower prevalence of osteoporosis (OR = 0.27; 95% CI, 0.14–0.53; *p* < 0.001) in model 3 in women (Supplementary Table S7). Model 1 and model 2 produced similar results to those of model 3 in both the men and women subgroup analyses. In the subgroup analysis stratified by smoking status, a significantly positive relationship was found between the blood Cd level and the prevalence of osteoporosis in the non-smoking subgroup, while no significant relationship was found in the smoking subgroup. Blood Se level showed a protective effect in the fourth quartile in both the smoking and non-smoking subgroups (Supplementary Tables S8, S9).

## Discussion

The present study explored the correlation between blood heavy metals and the higher prevalence of osteoporosis in a US population of middle-aged and elderly people. Based on the representative sample of the US population in NHANES (2013–2014 and 2017–2018), we found that Cd was independently associated with a higher prevalence of osteoporosis, while Se was independently associated with a lower prevalence of osteoporosis, and Pb, Hg, and Mn showed no statistically significant effect on the prevalence of osteoporosis.

Age, sex, and BMI are traditional risk factors for osteoporosis. The amount of bone in an individual peaks in young adulthood and one experiences subsequent loss with aging (19). Women lose bone more rapidly due to the lack of estrogen with aging, while men experience a slow loss of bone (20). Guidelines have recommended BMD screening for osteoporosis in women aged 65 years or older but clinical risk assessment tools for screening osteoporosis in younger women (21, 22). In the present study, the average age of participants in the osteoporosis group was older than that of participants in the non-osteoporosis group, which was consistent with that mentioned in previous studies. Studies demonstrated that aging may cause interstitial inflammation and fibrosis in renal tubuli, which are closely related to the excretion of heavy metals (23). A recent study revealed that the renal burden of Hg increases with age (24). Another study in southwestern China showed that higher blood heavy metals were found in older individuals compared with younger adults (25). A previous NHANES 2005–2006 study revealed a positive association between BMI and BMD (26), which was similar to the results of the present study. The NHANES 99-02 data showed that environmental exposure to Cd was negatively correlated with BMI (27). Another NHANES study reported that blood Hg levels were inversely correlated with BMI for adults (28). The present study showed similar results.

The relationship between blood Cd level and osteoporosis has been revealed in a small number of cross-sectional studies (29). Moreover, a recent study reported that Cd exposure was associated with an up to 23% increase in the incidence of osteoporosis, and the absolute cost of the burden of osteoporosis-related fractures caused by Cd is estimated to range between EUR€ 0.12 and 2.6 billion (30). Furthermore, Chung revealed that blood Cd concentrations of >1.0 μg/L and >0.5 μg/L were independent risk factors for incident osteoporosis in 243 participants and in 121 women, respectively, from the 2001 to 2002 Korea Genome and Epidemiology Study (31). The present study showed similar results. However, the sample size of the present study is relatively large and the research population is middle-aged and elderly populations, those who are more likely to have osteoporosis since it is a threat related to the aging population. A recent NHANES (2011–2018) study of young adults from 20 to 35 years revealed that blood Cd was independently negatively associated with lumbar BMD in women rather than men (32). However, a few studies explored the relationship between blood Cd and osteoporosis in men. This positive relationship was found in women in the present study but not in men. Moreover, the smoking subgroup was first discussed, the results revealed that smoking did not affect the relationship between blood Cd and the prevalence of osteoporosis. Furthermore, a recent study involving 488 women showed no correlation between blood Cd and osteoporosis (33). The potential mechanisms underlying the relationship between Cd and osteoporosis have been explored, including impairing the viability, proliferative ability, and osteogenic differentiation of bone marrow mesenchymal stem cells (BMMSCs) through the NF-κB and P2X7-PI3K-AKT signaling pathways (14, 34). Thus, the dysfunction of BMMSCs might be the main cause of Cd-related osteoporosis. *In vitro* studies showed that Cd stimulated osteoclastogenesis by increasing RANKL expression (35). Moreover, recent studies suggested that Cd induces bone osteoblast apoptosis *via* ROS (36). In addition, a further study demonstrated that Cd suppressed osteogenesis by inhibiting the Wnt/β-catenin pathway (37). *In vivo* studies further demonstrated that Cd induces a decreased expression of Runx2 and matrix proteins such as ALP, OCN, and COL1a2 (38). Another study found that Cd affected BMMSC differentiation by stimulating adipogenesis at the expense of osteoblastogenesis (39). Furthermore, other potential mechanisms, including Cd-related NF-κB and P2X7-PI3K-AKT signaling pathways, have recently been demonstrated to impair the viability, proliferative ability, and osteogenic differentiation of BMMSCs (14, 34).

Low blood Se status has been demonstrated to be correlated with skeletal disease, especially female the prevalence of osteoporosis (40, 41). A significant and positive relationship was observed between BMD and Se in a study involving 280 Spanish women (15). Beukhof et al. demonstrated that Se status was positively associated with BMD in a cohort of 387 healthy aging European men (42). In addition, some other studies revealed that Se was negatively associated with fractures induced by osteoporosis (43–45). Our findings were consistent with the findings of these studies. Furthermore, the present study also demonstrated that blood Se reduced the prevalence of osteoporosis in men. Moreover, this relationship was found in both the smoking group and the non-smoking subgroups. However, some previous studies did not suggest a relationship between Se and BMD in healthy women (46). In addition, no relationship between Se and osteoporosis has been reported in either an Asian or a European population (47–49). These results might be observed due to the differences in sample characteristics and loss of power (91–290 subjects). The potential mechanism for this viewpoint has been demonstrated *in vitro*, with evidence suggesting that Se enhances the osteoblastic differentiation of BMMSCs by downregulating the differentiation and formation of mature osteoclasts (50). Other *in vitro* studies have demonstrated that Se influences osteoblastic differentiation and subsequent bone resorption by regulating oxidative stress (51, 52). Previous studies revealed that inadequate levels of Se may alter bone metabolism and delay bone growth. *In vitro* studies showed that Se had a positive effect on osteoblastic differentiation and subsequent bone resorption by regulating oxidative stress (53). In addition, Wnt/LRP8/ApoER2 pathway was suggested as a fundamental intracellular Se transportation pathway for altering bone metabolism (54). Animal studies also found that bone metabolism changed with Se deprivation. Such effects were related to a decrease in GPX1 activity, blood concentrations of calcium, plasma insulin-like growth factor, pituitary growth hormone, and an increase in blood 1,25-dihydroxyvitamin D_3_, parathyroid hormone, and urinary calcium concentration (52). These changes were demonstrated to be associated with bone volume and BMD reduction, impairing bone microarchitecture (55).

The relationship between blood Pb and bone health has been reported in several epidemiological studies, but with inconsistent conclusions. A previous NHANES study (NHANES III) of adults aged ≥50 years showed that blood Pb was inversely correlated with BMD among white participants (56). In contrast, a significant inverse relationship between Pb and osteoporosis has been reported in the Korea National Health and Nutrition Examination Survey (2008–2011) (7). However, another previous study showed that blood Pb was not associated with BMD (57), which was consistent with that mentioned in the present study. In addition, we performed further analysis with gender and smoking subgroups and observed no relationship. A previous NHANES study (2005–2010) showed that a low blood Hg level was associated with an elevated risk of osteoporosis in young men (20–29 years) and women (30–39 years) (58). However, this relationship between the middle-aged population and the elderly population remains unclear. Our study showed that blood Hg was not associated with an increased prevalence of osteoporosis in low or medium blood Hg levels. However, a high blood Hg level was found to show a positive relationship with a higher prevalence of osteoporosis in men but not in women. High blood Hg levels were found to be associated with reduced BMD in the femur neck in the Korean National Health and Nutrition Examination Survey (2008–2010) (59). These inconsistent findings on the relationships of blood Hg with osteoporosis may be due to the heterogeneity between these studies. The relationship between blood Mn and osteoporosis remains unclear. A previous study of 91 elderly men showed no correlations between blood Mn level and BMD (47). Another research of 304 retired workers revealed that a high Mn exposure level was correlated with a higher risk of osteoporosis (60). No relationship was observed in the present study. This finding was similar to that of a previous study with a small sample size (61). However, further subgroup analysis showed that a higher blood Mn level was positively associated with a higher prevalence of osteoporosis in men and non-smoking subjects. These inconsistent findings may have contributed to the different biological specimens and the variation in Mn exposure levels.

The present studies showed that elevated blood Pb, Hg, and Mn levels were not correlated with a higher prevalence of osteoporosis. However, some studies showed either a positive or a negative relationship between these heavy metals and BMD (11, 62). The possible mechanism for the positive relationship may be attributed to oxidative stress-related toxicity in inhibiting the function of osteoblasts (63). Thus, it remains controversial as to whether the contents of Pb, Hg, and Mn can directly influence BMD and affect the pathogenesis of osteoporosis. The positive relationship between Cd exposure and a higher prevalence of lower BMD were proven in both animal models and human-based studies. These biological mechanisms are complex and are not fully understood. Excessive Cd exposure will reduce the production of calcitriol, decompose the collagen matrix in the bone, interfere with the mineralization of bone cells, inhibit the activity of osteoblasts, and stimulate the activity of osteoclasts, thus damaging bone health (10). The relationship between Se and bone health has been widely studied. As an essential component of selenoprotein, Se plays an important role in the maintenance of bone homeostasis through cell proliferation regulation and antioxidant protection (55). Further studies are worth being conducted to determine the relationship between Se exposure and osteoporosis and to explore the underlying mechanism.

However, there are several limitations to the present study. First, the present study used a cross-sectional design, and no causal inference between blood heavy metals and the prevalence of osteoporosis can be made. Second, although demographic, medical history, and lifestyle variables have been adjusted using logistic regression in the present study, confounding variables may still exist and affect the correction between blood heavy metals and the prevalence of osteoporosis. In addition, other variables, such as diet and hypertriglyceridemia, were not included in this analysis. Third, blood heavy metals were measured only one time and this type of measurement might not reflect a continuous exposure, thus measurement errors were inevitable. Finally, the number of participants in the osteoporosis group was relatively small and other treatment variables (vitamin D and bisphosphate) were not included in the analysis; further larger sample studies are needed to confirm the results. However, our study also carries some strengths. First, the present study was based on a relatively large dataset from the US population. Second, DXA is more accurate for the diagnosis of osteoporosis, and, finally, we performed further subgroup analysis on the relationship between blood heavy metals and the prevalence of osteoporosis.

## Conclusion

In conclusion, our study demonstrated that blood Cd level aggravated the prevalence of osteoporosis, while blood Se level could be a protective factor for the prevalence of osteoporosis among the US middle-aged and older populations. However, the results need to be confirmed in a prospective study.

## Data availability statement

The datasets presented in this study can be found in online repositories. The names of the repository/repositories and accession number(s) can be found in the article/Supplementary material.

## Ethics statement

The NHANES protocol is approved by the National Center for Health Statistics Institutional Review Board, and written informed consent is obtained. Written informed consent for participation was not required for this study in accordance with the national legislation and the institutional requirements.

## Author contributions

HWe and ZH: conception and design. XW: administrative support. HWa: provision of study materials or patients. XD and SZ: collection and assembly of data, data analysis, and interpretation. All authors wrote the manuscript and approved the final manuscript. All authors contributed to the article and approved the submitted version.
