# Supplementary material for: Relationship of blood heavy metals and osteoporosis among the middle-aged and elderly adults: A secondary analysis from NHANES 2013 to 2014 and 2017 to 2018
Source: Front Public Health. 2023 Mar 14;11:1045020. doi: 10.3389/fpubh.2023.1045020 (PMC10043376; doi:10.3389/fpubh.2023.1045020)
Supplement: Supplementary file 1 [file Data_Sheet_1.docx]

| **Table S1 Characteristics of participants based on blood Pb in the enrolled population of NHANES** | | | | | | | | | |  |
| --- | --- | --- | --- | --- | --- | --- | --- | --- | --- | --- |
| **Characteristic** | **Total (N = 1777)** | | **Q1(N = 442)** | **Q2 (N = 441)** | **Q3(N = 449)** | **Q4 (N = 445)** | ***F/χ^2^*** |  | ***P*-value** | |
| Age (Weighted years, mean ± SD） | 58.9 ± 0.4 | | 56.6 ± 0.9 | 59.7± 0.7 | 59.7 ± 0.8 | 60.3 ± 0.6 | 10.14 |  | < 0.001 | |
| Gender, No. (Weighted %)^a^ | | |  |  |  |  | 27.51 |  | 0.002 | |
| Male | | 903 (50.4) | 194 (41.0) | 223 (55.1) | 235(50.7) | 251 (56.5) |  |  |  | |
| Female | | 874 (49.6) | 248 (59.0) | 218 (44.9) | 214 (49.3) | 194 (43.4) |  |  |  | |
| Race, No. (Weighted %)^a^ | | |  |  |  |  | 25.77 |  | 0.222 | |
| Mexican | | 218 (8.8) | 53 (7.9) | 60 (11.6) | 60 (9.0) | 45 (6.3) |  |  |  | |
| Other Hispanic | | 165 (6.0) | 53 (8.4) | 32 (4.1) | 39 (5.9) | 41 (5.5) |  |  |  | |
| Non-Hispanic White | | 788 (61.8) | 194 (61.2) | 214 (64.5) | 185 (60.3) | 195 (60.7) |  |  |  | |
| Non-Hispanic Black | | 351 (13.4) | 84 (12.4) | 76 (11.8) | 98 (15.7) | 93 (14.1) |  |  |  | |
| Other Race | | 255 (10.0) | 58 (10.1) | 59 (8.0) | 67 (9.1) | 71 (13.4) |  |  |  | |
| Education, No. (Weighted %)^a^ | | |  |  |  |  | 25.18 |  | 0.104 | |
| Less than 9th grade | | 209 (9.5) | 55 (10.1) | 47 (9.9) | 50 (7.5) | 57 (10.5) |  |  |  | |
| 9-11 grade | | 503 (25.1) | 117 (23.3) | 101 (21.1) | 151 (31.3) | 134 (25.5) |  |  |  | |
| High school graduate or equivalent | | 423 (24.0) | 95 (23.3) | 110 (23.9) | 102 (26.1) | 116 (22.7) |  |  |  | |
| Some college or AA degree | | 304 (17.5) | 83 (19.1) | 87 (17.7) | 63 (13.1) | 71 (20.3) |  |  |  | |
| College graduate or above | | 338 (23.9) | 92 (20.2) | 96 (27.4) | 83 (22.0) | 67 (21.0) |  |  |  | |
| BMI, No. (Weighted %)^a^ | | |  |  |  |  | 60.11 |  | < 0.001 | |
| Normal (< 25) | | 442 (23.5) | 86 (20.2) | 85 (17.4) | 122 (27.2) | 149 (31.4) |  |  |  | |
| Overweight (25-30) | | 662 (38.3) | 143 (31.2) | 182 (39.4) | 170 (38.7) | 167 (41.2) |  |  |  | |
| Obesity (> 30) | | 673 (38.2) | 213 (48.6) | 174 (43.2) | 157 (34.1) | 129 (27.4) |  |  |  | |
| Smoking status, No. (Weighted %)^a^ | | |  |  |  |  | 67.00 |  | < 0.001 | |
| Yes | | 382 (18.9) | 51 (11.2) | 75 (13.1) | 117 (26.4) | 139 (28.4) |  |  |  | |
| No | | 1395 (81.1) | 391 (88.8) | 366 (86.9) | 332 (73.6) | 306 (71.6) |  |  |  | |
| Physical activity, No. (Weighted %)^a^ | | |  |  |  |  | 6.25 |  | 0.338 | |
| Yes | | 350 (20.8) | 77 (18.8) | 79 (19.7) | 88 (20.4) | 106 (25.4) |  |  |  | |
| No | | 1427 (79.2) | 365 (81.2) | 362 (80.3) | 361 (79.6) | 339 (74.6) |  |  |  | |
| Diabetes, No. (Weighted %)^a^ | | |  |  |  |  | 33.49 |  | < 0.001 | |
| Yes | | 362 (16.9) | 110 (23.5) | 100 (18.8) | 79 (11.0) | 73 (12.0) |  |  |  | |
| No | | 1415 (83.1) | 332 (76.5) | 341 (81.2) | 370 (89.0) | 372 (88.0) |  |  |  | |
| Hypertension, No. (Weighted %)^a^ | | |  |  |  |  | 4.16 |  | 0.588 | |
| Yes | | 878 (45.0) | 209 (48.1) | 216 (43.6) | 221 (42.0) | 232 (46.2) |  |  |  | |
| No | | 899 (55.0) | 233 (51.9) | 225 (56.4) | 228 (58.0) | 213 (53.8) |  |  |  | |
| Alcohol Consumption, No. (Weighted %)^a^ | | | | | | | 0.55 |  | 0.961 | |
| ≥ 12 times per year | | 247 (13.8) | 63 (14.4) | 70 (13.9) | 57 (13.9) | 57 (12.7) |  |  |  | |
| < 12 times per year | | 1530 (86.2) | 379 (85.6) | 371 (86.1) | 392 (86.1) | 388 (87.3) |  |  |  | |
| Exposure to secondhand smoke, No. (Weighted %)^a^ | | | | | | | 36.56 |  | < 0.001 | |
| Yes | | 452 (22.5) | 80 (16.6) | 92 (18.6) | 126 (25.7) | 154 (32.2) |  |  |  | |
| No | | 1325 (77.5) | 362 (83.4) | 349 (81.4) | 323 (74.3) | 291 (67.8) |  |  |  | |
| Sedentary behavior, No. (Weighted %)^a^ | | | | | | | 4.54 |  | 0.491 | |
| Yes | | 1025 (60.3) | 250 (60.9) | 268 (62.8) | 257 (56.1) | 250 (61.0) |  |  |  | |
| No | | 752 (39.7) | 192 (39.1) | 173 (37.2) | 192 (43.9) | 195 (39.0) |  |  |  | |
| Arthritis, No. (Weighted %)^a^ | | | | | | | 2.58 |  | 0.752 | |
| Yes | | 677 (39.4) | 151 (37.4) | 171 (38.2) | 171 (41.2) | 184 (41.7) |  |  |  | |
| No | | 1100 (60.6) | 291 (62.6) | 270 (61.8) | 278 (58.8) | 261 (58.3) |  |  |  | |
| Thyroid problems, No. (Weighted %)^a^ | | | | | | | 14.49 |  | 0.051 | |
| Yes | | 253 (16.4) | 78 (20.9) | 59 (12.6) | 65 (17.6) | 51 (13.9) |  |  |  | |
| No | | 1774 (83.6) | 364 (79.1) | 382 (87.4) | 384 (82.4) | 394 (86.1) |  |  |  | |
| Hypercholesterolemia, No. (Weighted %)^a^ | | | | | | | 22.18 |  | 0.003 | |
| Yes | | 763 (44.4) | 175 (38.4) | 190 (41.4) | 185 (47.1) | 213 (53.3) |  |  |  | |
| No | | 1014 (55.6) | 267 (61.6) | 251 (58.6) | 264 (52.9) | 232 (46.7) |  |  |  | |
| GFR, No. (Weighted %)^a^ | | | | | | | 35.48 |  | 0.003 | |
| < 60 mL/min/1.73 m^2^ | | 265 (13.2) | 30 (7.7) | 61 (13.6) | 80 (15.0) | 94 (18.3) |  |  |  | |
| 60-90 mL/min/1.73 m^2^ | | 810 (47.7) | 174(45.1) | 200 (47.4) | 221 (48.7) | 215 (50.2) |  |  |  | |
| ≥ 90 mL/min/1.73 m^2^ | | 702 (39.1) | 238 (47.2) | 180 (39.0) | 148 (36.3) | 136 (31.5) |  |  |  | |
| Annual household income, No. (Weighted %)^a^ | | | | | | | 50.54 |  | 0.003 | |
| $0 to $19,999 | | 327 (11.2) | 67 (8.4) | 72 (10.5) | 86 (12.6) | 102 (14.6) |  |  |  | |
| $20,000, to $34,999 | | 332 (13.8) | 70 (10.7) | 63 (9.8) | 89 (15.5) | 110 (21.2) |  |  |  | |
| $35,000 to $74,999 | | 421 (24.3) | 97 (22.0) | 118 (28.2) | 116 (24.7) | 90 (21.9) |  |  |  | |
| $75,000 and Over | | 697 (50.7) | 208 (5.9) | 188 (51.5) | 158(47.2) | 143 (42.3) |  |  |  | |

^a^ Numbers of participants are unweighted. All percentage estimates are weighted.

| **Table S2 Characteristics of participants based on blood Cd in the enrolled population of NHANES** | | | | | | | | | |  |
| --- | --- | --- | --- | --- | --- | --- | --- | --- | --- | --- |
| **Characteristic** | **Total (N = 1777)** | | **Q1(N = 425)** | **Q2 (N = 458)** | **Q3(N = 445)** | **Q4 (N = 449)** | ***F/χ^2^*** |  | ***P*-value** | |
| Age (Weighted years, mean ± SD） | 58.9 ± 0.4 | | 57.1 ± 0.8 | 60.0 ± 0.8 | 60.9 ± 0.6 | 58.0 ± 0.7 | 10.11 |  | < 0.001 | |
| Gender, No. (Weighted %)^a^ | | |  |  |  |  | 49.73 |  | < 0.001 | |
| Male | | 903 (50.4) | 251 (62.4) | 241 (50.2) | 204 (43.5) | 207 (41.2) |  |  |  | |
| Female | | 874 (49.6) | 174 (37.6) | 217 (49.8) | 241 (56.5) | 242 (58.8) |  |  |  | |
| Race, No. (Weighted %)^a^ | | |  |  |  |  | 22.16 |  | 0.359 | |
| Mexican | | 218 (8.8) | 57 (9.9) | 69 (8.8) | 52 (8.3) | 40 (8.0) |  |  |  | |
| Other Hispanic | | 165 (6.0) | 45 (5.0) | 39 (5.1) | 43 (7.0) | 38 (7.6) |  |  |  | |
| Non-Hispanic White | | 788 (61.8) | 200 (64.6) | 245 (65.0) | 186 (6.1) | 187 (54.9) |  |  |  | |
| Non-Hispanic Black | | 351 (13.4) | 83 (12.3) | 78 (13.3) | 86 (12.1) | 104 (16.2) |  |  |  | |
| Other Race | | 255 (10.0) | 40 (8.2) | 57 (7.7) | 78 (11.9) | 80 (16.3) |  |  |  | |
| Education, No. (Weighted %)^a^ | | |  |  |  |  | 45.12 |  | 0.033 | |
| Less than 9th grade | | 209 (9.5) | 42 (9.1) | 50 (8.0) | 69 (12.3) | 48 (9.2) |  |  |  | |
| 9-11 grade | | 503 (25.1) | 109 (21.4) | 123 (23.4) | 122 (25.9) | 149 (31.3) |  |  |  | |
| High school graduate or equivalent | | 423 (24.0) | 101 (24.8) | 96 (21.4) | 97 (20.8) | 129 (29.5) |  |  |  | |
| Some college or AA degree | | 304 (17.5) | 80 (18.6) | 81 (18.0) | 70 (16.9) | 73 (16.2) |  |  |  | |
| College graduate or above | | 338 (23.9) | 93 (26.1) | 108 (29.2) | 87 (24.1) | 50 (13.8) |  |  |  | |
| BMI, No. (Weighted %)^a^ | | |  |  |  |  | 58.36 |  | < 0.001 | |
| Normal (< 25) | | 442 (23.5) | 74 19.3) | 87 (17.8) | 109 (24.6) | 172 (35.3) |  |  |  | |
| Overweight (25-30) | | 662 (38.3) | 148 (34.3) | 178 (42.7) | 177 (41.0) | 159 (35.4) |  |  |  | |
| Obesity (> 30) | | 673 (38.2) | 203 (46.4) | 193 (39.5) | 159 (34.4) | 118 (29.3) |  |  |  | |
| Smoking status, No. (Weighted %)^a^ | | |  |  |  |  | 716.46 |  | < 0.001 | |
| Yes | | 382 (18.9) | 12 (1.8) | 25 (4.4) | 59 (14.2) | 286 (65.8) |  |  |  | |
| No | | 1395 (81.1) | 413 (98.2) | 433 (95.6) | 386 (85.8) | 163 (34.2) |  |  |  | |
| Physical activity, No. (Weighted %)^a^ | | |  |  |  |  | 17.84 |  | 0.018 | |
| Yes | | 350 (20.8) | 91 (25.2) | 77 (16.8) | 82 (16.6) | 100 (24.3) |  |  |  | |
| No | | 1427 (79.2) | 334 (74.8) | 381 (83.2) | 363 (83.4) | 349 (75.7) |  |  |  | |
| Diabetes, No. (Weighted %)^a^ | | |  |  |  |  | 14.84 |  | 0.034 | |
| Yes | | 362 (16.9) | 105 (19.7) | 101 (20.0) | 87 (13.3) | 69 (12.5) |  |  |  | |
| No | | 1415 (83.1) | 320 (80.3) | 357 (80.0) | 358 (86.7) | 380 (87.5) |  |  |  | |
| Hypertension, No. (Weighted %)^a^ | | |  |  |  |  | 0.18 |  | 0.988 | |
| Yes | | 878 (45.0) | 204 (45.8) | 231 (44.6) | 212 (44.8) | 231 (44.8) |  |  |  | |
| No | | 899 (55.0) | 221 (54.2) | 227 (55.4)) | 233 (55.2) | 218 (55.2) |  |  |  | |
| Alcohol Consumption, No. (Weighted %)^a^ | | | | | | | 4.72 |  | 0.706 | |
| ≥ 12 times per year | | 247 (13.8) | 45 (11.1) | 69 (14.6) | 72 (15.6) | 61 (14.7) |  |  |  | |
| < 12 times per year | | 1530 (86.2) | 380 (88.9) | 389 (85.4) | 373 (84.4) | 388 (85.3) |  |  |  | |
| Exposure to secondhand smoke, No. (Weighted %)^a^ | | | | | | | 328.90 |  | < 0.001 | |
| Yes | | 452 (22.5) | 58 (14.1) | 53 (9.2) | 86 (17.4) | 255 (56.6) |  |  |  | |
| No | | 1325 (77.5) | 367 (85.9) | 405 (90.8) | 359 (82.6) | 194 (43.4) |  |  |  | |
| Sedentary behavior, No. (Weighted %)^a^ | | | | | | | 5.35 |  | 0.562 | |
| Yes | | 1025 (60.3) | 249 (63.4) | 248 (56.7) | 265 (61.7) | 263 (59.3) |  |  |  | |
| No | | 752 (39.7) | 176 (36.6) | 210 (43.3) | 180 (38.3) | 186 (40.7) |  |  |  | |
| Arthritis, No. (Weighted %)^a^ | | | | | | | 19.50 |  | 0.008 | |
| Yes | | 677 (39.4) | 144 (31.6) | 158 (41.5) | 184 (42.4) | 191 (44.3) |  |  |  | |
| No | | 1100 (60.6) | 281 (68.4) | 300 (58.5) | 261 (57.6) | 258 (55.7) |  |  |  | |
| Thyroid problems, No. (Weighted %)^a^ | | | | | | | 16.00 |  | 0.052 | |
| Yes | | 253 (16.4) | 47 (11.1) | 66 (17.4) | 76 (20.5) | 64 (18.2) |  |  |  | |
| No | | 1774 (83.6) | 378 (88.8) | 392 (82.6) | 369 (79.5) | 385 (81.8) |  |  |  | |
| Hypercholesterolemia, No. (Weighted %)^a^ | | | | | | | 18.33 |  | 0.016 | |
| Yes | | 763 (44.4) | 162 (38.9) | 191 (42.8) | 213 (52.9) | 197 (45.3) |  |  |  | |
| No | | 1014 (55.6) | 263 (61.1) | 267 (57.2) | 232 (47.1) | 252 (54.7) |  |  |  | |
| GFR, No. (Weighted %)^a^ | | | | | | | 18.45 |  | 0.183 | |
| < 60 mL/min/1.73 m^2^ | | 265 (13.2) | 71 (11.7) | 67 (14.3) | 72 (12.8) | 55 (14.2) |  |  |  | |
| 60-90 mL/min/1.73 m^2^ | | 810 (47.7) | 191 (45.8) | 217 (51.3) | 211 (52.3) | 191 (40.8) |  |  |  | |
| ≥ 90 mL/min/1.73 m^2^ | | 702 (39.1) | 179 (42.5) | 169 (34.4) | 167 (34.8) | 187 (45.0) |  |  |  | |
| Annual household income, No. (Weighted %)^a^ | | | | | | | 148.19 |  | < 0.001 | |
| $0 to $19,999 | | 327 (11.2) | 67 (7.3) | 62 (6.5) | 68(13.0) | 130 (21.2) |  |  |  | |
| $20,000, to $34,999 | | 332 (13.8) | 64 (8.4) | 65 (10.1) | 89 (16.2) | 114 (23.4) |  |  |  | |
| $35,000 to $74,999 | | 421 (24.3) | 95 (24.0) | 113 (23.7) | 114 (23.7) | 99 (26.3) |  |  |  | |
| $75,000 and Over | | 697 (50.7) | 199 (60.3) | 218 (59.7) | 174 (47.1) | 106 (29.1) |  |  |  | |

^a^ Numbers of participants are unweighted. All percentage estimates are weighted.

| **Table S3 Characteristics of participants based on blood Hg in the enrolled population of NHANES** | | | | | | | | | |  |
| --- | --- | --- | --- | --- | --- | --- | --- | --- | --- | --- |
| **Characteristic** | **Total (N = 1777)** | | **Q1(N = 435)** | **Q2 (N = 447)** | **Q3(N = 448)** | **Q4 (N = 447)** | ***F/χ^2^*** |  | ***P*-value** | |
| Age (Weighted years, mean ± SD） | 58.9 ± 0.4 | | 58.1 ± 0.6 | 58.5 ± 0.8 | 59.5 ± 0.8 | 59.6 ± 0.8 | 1.82 |  | 0.141 | |
| Gender, No. (Weighted %)^a^ | | |  |  |  |  | 17.44 |  | 0.106 | |
| Male | | 903 (50.4) | 203 (45.6) | 242 (54.7) | 227 (47.1) | 231 (54.2) |  |  |  | |
| Female | | 874 (49.6) | 232 (54.4) | 205 (45.3) | 221 (52.9) | 216 (45.8) |  |  |  | |
| Race, No. (Weighted %)^a^ | | |  |  |  |  | 31.68 |  | 16.87 | |
| Mexican | | 218 (8.8) | 69 (9.9) | 62 (8.5) | 47 (10.1) | 40 (6.8) |  |  |  | |
| Other Hispanic | | 165 (6.0) | 30 (6.4) | 44 (4.7) | 49 (7.9) | 42 (54.1) |  |  |  | |
| Non-Hispanic White | | 788 (61.8) | 208 (61.4) | 204 (67.9) | 204 (60.5) | 172 (57.4) |  |  |  | |
| Non-Hispanic Black | | 351 (13.4) | 75 (12.3) | 83 (10.9) | 112 (14.1) | 81 (16.2) |  |  |  | |
| Other Race | | 255 (10.0) | 53 (10.0) | 54 (10.0) | 36 (7.4) | 112 (14.5) |  |  |  | |
| Education, No. (Weighted %)^a^ | | |  |  |  |  | 54.86 |  | 0.019 | |
| Less than 9th grade | | 209 (9.5) | 53 (9.3) | 62 (9.5) | 48 (8.7) | 46 (10.5) |  |  |  | |
| 9-11 grade | | 503 (25.1) | 142 (32.3) | 126 (24.3) | 130 (24.0) | 105 (20.0) |  |  |  | |
| High school graduate or equivalent | | 423 (24.0) | 120 (27.0) | 110 (23.1) | 96 (21.8) | 97 (24.2) |  |  |  | |
| Some college or AA degree | | 304 (17.5) | 70 (15.4) | 82 (22.1) | 81 (19.2) | 71 (13.4) |  |  |  | |
| College graduate or above | | 338 (23.9) | 50 (15.9) | 67 (21.0) | 93 (26.3) | 128 (31.9) |  |  |  | |
| BMI, No. (Weighted %)^a^ | | |  |  |  |  | 29.46 |  | 0.010 | |
| Normal (< 25) | | 442 (23.5) | 100 (23.1) | 97 (18.1) | 107 (22.4) | 138 (30.1) |  |  |  | |
| Overweight (25-30) | | 662 (38.3) | 143 (33.2) | 159 (40.0) | 185 (42.6) | 175 (37.2) |  |  |  | |
| Obesity (> 30) | | 673 (38.2) | 192 (43.7) | 191 (41.9) | 156 (35.1) | 134 (32.7) |  |  |  | |
| Smoking status, No. (Weighted %)^a^ | | |  |  |  |  | 35.62 |  | < 0.001 | |
| Yes | | 382 (18.9) | 116 (23.7) | 110 (22.2) | 103 (20.4) | 53 (9.6) |  |  |  | |
| No | | 1395 (81.1) | 319 (76.3) | 337 (77.8) | 345 (79.6) | 394 (90.4) |  |  |  | |
| Physical activity, No. (Weighted %)^a^ | | |  |  |  |  | 1.30 |  | 0.908 | |
| Yes | | 350 (20.8) | 101 (22.2) | 81 (19.1) | 89 (20.8) | 79 (21.1) |  |  |  | |
| No | | 1427 (79.2) | 334 (77.8) | 366 (80.9) | 359 (79.2) | 368 (78.9) |  |  |  | |
| Diabetes, No. (Weighted %)^a^ | | |  |  |  |  | 3.86 |  | 0.552 | |
| Yes | | 362 (16.9) | 104 (19.8) | 95 (16.7) | 78 (16.0) | 85 (15.2) |  |  |  | |
| No | | 1415 (83.1) | 331 (80.2) | 352 (83.3) | 370 (84.0) | 362 (84.8) |  |  |  | |
| Hypertension, No. (Weighted %)^a^ | | |  |  |  |  | 7.69 |  | 0.377 | |
| Yes | | 878 (45.0) | 221 (45.2) | 223 (49.9) | 218 (44.6) | 216 (40.6) |  |  |  | |
| No | | 899 (55.0) | 214 (54.8) | 224 (50.1) | 230 (55.4) | 231 (59.4) |  |  |  | |
| Alcohol Consumption, No. (Weighted %)^a^ | | | | | | | 3.98 |  | 0.678 | |
| ≥ 12 times per year | | 247 (13.8) | 64 (16.4) | 55 (12.2) | 75 (14.1) | 53 (12.6) |  |  |  | |
| < 12 times per year | | 1530 (86.2) | 371 (83.6) | 392 (87.8) | 373 (85.9) | 394 (87.4) |  |  |  | |
| Exposure to secondhand smoke, No. (Weighted %)^a^ | | | | | | | 23.17 |  | 0.055 | |
| Yes | | 452 (22.5) | 139 (29.1) | 119 (22.9) | 116 (22.8) | 78 (15.6) |  |  |  | |
| No | | 1325 (77.5) | 296 (70.9) | 328 (77.2) | 332 (77.2) | 369 (84.4) |  |  |  | |
| Sedentary behavior, No. (Weighted %)^a^ | | | | | | | 3.92 |  | 0.675 | |
| Yes | | 1025 (60.3) | 254 (63.3) | 262 (61.2) | 259 (57.0) | 250 (59.8) |  |  |  | |
| No | | 752 (39.7) | 181 (36.7) | 185 (38.8) | 189 (43.0) | 197 (40.2) |  |  |  | |
| Arthritis, No. (Weighted %)^a^ | | | | | | | 1.24 |  | 0.885 | |
| Yes | | 677 (39.4) | 162 (39.1) | 198 (41.3) | 167 (39.6) | 150 (37.7) |  |  |  | |
| No | | 1100 (60.6) | 273 (60.9) | 249 (58.7) | 281 (60.4) | 297 (62.3) |  |  |  | |
| Thyroid problems, No. (Weighted %)^a^ | | | | | | | 28.68 |  | < 0.001 | |
| Yes | | 253 (16.4) | 76 (22.0) | 62 (14.3) | 72 (19.6) | 43 (9.8) |  |  |  | |
| No | | 1774 (83.6) | 359 (78.0) | 385 (85.7) | 376 (80.4) | 404 (90.1) |  |  |  | |
| Hypercholesterolemia, No. (Weighted %)^a^ | | | | | | | 6.89 |  | 0.321 | |
| Yes | | 763 (44.4) | 167 (41.9) | 175 (40.8) | 209 (46.2) | 212 (48.4) |  |  |  | |
| No | | 1014 (55.6) | 268 (58.1) | 272 (59.2) | 239 (53.8) | 235 (51.6) |  |  |  | |
| GFR, No. (Weighted %)^a^ | | | | | | | 15.03 |  | 0.232 | |
| < 60 mL/min/1.73 m^2^ | | 265 (13.2) | 63 (12.4) | 70 (13.6) | 71 (15.3) | 61 (11.5) |  |  |  | |
| 60-90 mL/min/1.73 m^2^ | | 810 (47.7) | 201 (49.1) | 186 (40.9) | 209 (49.1) | 214 (51.3) |  |  |  | |
| ≥ 90 mL/min/1.73 m^2^ | | 702 (39.1) | 171 (38.5) | 191 (45.5) | 168 (35.6) | 172 (37.2) |  |  |  | |
| Annual household income, No. (Weighted %)^a^ | | | | | | | 148.55 |  | < 0.001 | |
| $0 to $19,999 | | 327 (11.2) | 124 (19.1) | 98 (14.9) | 64 (7.0) | 41 (4.6) |  |  |  | |
| $20,000, to $34,999 | | 332 (13.8) | 97 (16.3) | 98 (15.4) | 81 (13.5) | 56 (10.0) |  |  |  | |
| $35,000 to $74,999 | | 421 (24.3) | 100 (29.4) | 117 (28.4) | 112 (24.7) | 92 (15.1) |  |  |  | |
| $75,000 and Over | | 697 (50.7) | 114 (35.2) | 134 (41.3) | 191 (54.8) | 258 (70.3) |  |  |  | |

^a^ Numbers of participants are unweighted. All percentage estimates are weighted.

| **Table S4 Characteristics of participants based on blood Se in the enrolled population of NHANES** | | | | | | | | | |  |
| --- | --- | --- | --- | --- | --- | --- | --- | --- | --- | --- |
| **Characteristic** | **Total (N = 1777)** | | **Q1(N = 444)** | **Q2 (N = 444)** | **Q3(N = 444)** | **Q4 (N = 445)** | ***F/χ^2^*** |  | ***P*-value** | |
| Age (Weighted years, mean ± SD） | 58.9 ± 0.4 | | 60.5 ± 1.0 | 59.1 ± 0.7 | 58.5 ± 0.9 | 57.9 ± 0.7 | 4.19 |  | 0.006 | |
| Gender, No. (Weighted %)^a^ | | |  |  |  |  | 16.90 |  | 0.066 | |
| Male | | 903 (50.4) | 218 (44.8) | 195 (45.8) | 240 (55.7) | 250 (54.4) |  |  |  | |
| Female | | 874 (49.6) | 226 (55.2) | 249 (54.2) | 204 (44.3) | 195 (45.6) |  |  |  | |
| Race, No. (Weighted %)^a^ | | |  |  |  |  | 19.66 |  | 0.712 | |
| Mexican | | 218 (8.8) | 46 (9.2) | 59 (6.9) | 59 (8.0) | 54 (11.2) |  |  |  | |
| Other Hispanic | | 165 (6.0) | 42 (5.7) | 40 (7.7) | 47 (5.2) | 36 (5.6) |  |  |  | |
| Non-Hispanic White | | 788 (61.8) | 199 (59.3) | 205 (64.0) | 194 (65.3) | 190 (58.4) |  |  |  | |
| Non-Hispanic Black | | 351 (13.4) | 95 (15.6) | 83 (12.0) | 81 (10.7) | 92 (15.4) |  |  |  | |
| Other Race | | 255 (10.0) | 62 (10.2) | 57 (9.4) | 63 (10.8) | 73 (9.4) |  |  |  | |
| Education, No. (Weighted %)^a^ | | |  |  |  |  | 51.01 |  | 0.013 | |
| Less than 9th grade | | 209 (9.5) | 63 (14.2) | 59 (9.9) | 42 (6.3) | 45 (8.2) |  |  |  | |
| 9-11 grade | | 503 (25.1) | 156 (30.5) | 128 (22.1) | 118 (23.6) | 101 (24.6) |  |  |  | |
| High school graduate or equivalent | | 423 (24.0) | 107 (22.4) | 104 (29.6) | 98 (20.8) | 114 (23.1) |  |  |  | |
| Some college or AA degree | | 304 (17.5) | 56 (10.8) | 73 (16.8) | 82 (21.9) | 93 (19.9) |  |  |  | |
| College graduate or above | | 338 (23.9) | 62 (22.0) | 80 (21.6) | 104 (27.4) | 92 (24.2) |  |  |  | |
| BMI, No. (Weighted %)^a^ | | |  |  |  |  | 31.13 |  | 0.008 | |
| Normal (< 25) | | 442 (23.5) | 135 (31.1) | 108 (24.1) | 98 (20.9) | 101 (18.8) |  |  |  | |
| Overweight (25-30) | | 662 (38.3) | 149 (33.2) | 152 (35.3) | 173 (37.7) | 188 (46.1) |  |  |  | |
| Obesity (> 30) | | 673 (38.2) | 160 (35.6) | 184 (40.6) | 173 (41.4) | 156 (35.1) |  |  |  | |
| Smoking status, No. (Weighted %)^a^ | | |  |  |  |  | 23.57 |  | 0.001 | |
| Yes | | 382 (18.9) | 129 (25.7) | 104 (20.9) | 67 (13.6) | 82 (16.4) |  |  |  | |
| No | | 1395 (81.1) | 315 (74.3) | 340 (79.1) | 377 (86.4) | 363 (83.6) |  |  |  | |
| Physical activity, No. (Weighted %)^a^ | | |  |  |  |  | 15.32 |  | 0.053 | |
| Yes | | 350 (20.8) | 81 (19.3) | 101 (24.7) | 93 (23.8) | 75 (18.5) |  |  |  | |
| No | | 1427 (79.2) | 363 (80.7) | 343 (75.3) | 351 (76.2) | 370 (81.5) |  |  |  | |
| Diabetes, No. (Weighted %)^a^ | | |  |  |  |  | 4.38 |  | 0.555 | |
| Yes | | 362 (16.9) | 92 (17.1) | 79 (13.8) | 92 (18.0) | 99 (15.8) |  |  |  | |
| No | | 1415 (83.1) | 352 (82.9) | 365 (86.2) | 352 (82.0) | 346 (84.2) |  |  |  | |
| Hypertension, No. (Weighted %)^a^ | | |  |  |  |  | 7.15 |  | 0.149 | |
| Yes | | 878 (45.0) | 221 (49.3) | 218 (44.1) | 215 (40.6) | 224 (46.6) |  |  |  | |
| No | | 899 (55.0) | 223 (50.7) | 226 (55.9) | 229 (59.4) | 221 (53.4) |  |  |  | |
| Alcohol Consumption, No. (Weighted %)^a^ | | | | | | | 5.15 |  | 0.471 | |
| ≥ 12 times per year | | 247 (13.8) | 126 (16.2) | 121 (12.8) | 91 (15.1) | 114 (11.5) |  |  |  | |
| < 12 times per year | | 1530 (86.2) | 318 (83.8) | 323 (87.2) | 353 (84.9) | 331 (88.5) |  |  |  | |
| Exposure to secondhand smoke, No. (Weighted %)^a^ | | | | | | | 9.66 |  | 0.160 | |
| Yes | | 452 (22.5) | 421 (23.9) | 31 (25.5) | 421 (17.4) | 31 (23.4) |  |  |  | |
| No | | 1325 (77.5) | 1241 (76.1) | 84 (74.5) | 1241 (82.6) | 84 (76.6) |  |  |  | |
| Sedentary behavior, No. (Weighted %)^a^ | | | | | | | 0.74 |  | 0.941 | |
| Yes | | 1025 (60.3) | 260 (62.1) | 255 (59.9) | 256 (60.0) | 254 (59.4) |  |  |  | |
| No | | 752 (39.7) | 184 (37.9) | 189 (40.1) | 188 (40.0) | 191 (40.6) |  |  |  | |
| Arthritis, No. (Weighted %)^a^ | | | | | | | 21.18 |  | 0.002 | |
| Yes | | 677 (39.4) | 191 (48.0) | 179 (41.0) | 145 (34.1) | 162 (35.6) |  |  |  | |
| No | | 1100 (60.6) | 253 (52.0) | 265 (49.0) | 299 (65.9) | 283 (64.4) |  |  |  | |
| Thyroid problems, No. (Weighted %)^a^ | | | | | | | 12.13 |  | 0.242 | |
| Yes | | 253 (16.4) | 191 (21.6) | 179 (16.9) | 145 (13.7) | 162 (14.1) |  |  |  | |
| No | | 1774 (83.6) | 253 (78.4) | 265 (83.1) | 299 (86.3) | 283 (85.9) |  |  |  | |
| Hypercholesterolemia, No. (Weighted %)^a^ | | | | | | | 50.37 |  | < 0.001 | |
| Yes | | 763 (44.4) | 149 (29.8) | 189 (45.0) | 212 (47.8) | 213 (52.8) |  |  |  | |
| No | | 1014 (55.6) | 295 (70.2) | 255 (55.0) | 232 (52.1) | 232 (47.2) |  |  |  | |
| GFR, No. (Weighted %)^a^ | | | | | | | 14.21 |  | 0.179 | |
| < 60 mL/min/1.73 m^2^ | | 265 (13.2) | 74 (16.1) | 76 (12.2) | 60 (11.9) | 55 (13.1) |  |  |  | |
| 60-90 mL/min/1.73 m^2^ | | 810 (47.7) | 201 (49.0) | 189 (47.9) | 211 (51.8) | 209 (42.4) |  |  |  | |
| ≥ 90 mL/min/1.73 m^2^ | | 702 (39.1) | 169 (34.9) | 179 (39.9) | 173 (36.3) | 181 (44.5) |  |  |  | |
| Annual household income, No. (Weighted %)^a^ | | | | | | | 34.43 |  | 0.019 | |
| $0 to $19,999 | | 327 (11.2) | 114 (16.6) | 88 (14.2) | 59 (6.3) | 66 (8.7) |  |  |  | |
| $20,000, to $34,999 | | 332 (13.8) | 84 (14.4) | 81 (13.8) | 86 (13.7) | 81 (13.3) |  |  |  | |
| $35,000 to $74,999 | | 421 (24.3) | 104 (22.2) | 101 (26.0) | 106 (23.6) | 110 (25.1) |  |  |  | |
| $75,000 and Over | | 697 (50.7) | 142 (46.8) | 174 (46.0) | 193 (56.4) | 188 (52.9) |  |  |  | |

^a^ Numbers of participants are unweighted. All percentage estimates are weighted.

| **Table S5 Characteristics of participants based on blood Mn in the enrolled population of NHANES** | | | | | | | | | |  |
| --- | --- | --- | --- | --- | --- | --- | --- | --- | --- | --- |
| **Characteristic** | **Total (N = 1777)** | | **Q1(N = 443)** | **Q2 (N = 444)** | **Q3(N = 445)** | **Q4 (N = 445)** | ***F/χ^2^*** |  | ***P*-value** | |
| Age (Weighted years, mean ± SD） | 58.9 ± 0.4 | | 58.1 ± 0.6 | 59.6± 0.8 | 59.1 ± 0.9 | 58.9 ± 0.9 | 1.30 |  | 0.273 | |
| Gender, No. (Weighted %)^a^ | | |  |  |  |  | 14.49 |  | 0.047 | |
| Male | | 903 (50.4) | 255 (57.6) | 236 (49.6) | 218 (49.2) | 194 (44.7) |  |  |  | |
| Female | | 874 (49.6) | 188 (42.4) | 208 (50.4) | 227 (50.8) | 251 (55.3) |  |  |  | |
| Race, No. (Weighted %)^a^ | | |  |  |  |  | 30.99 |  | 0.183 | |
| Mexican | | 218 (8.8) | 43 (8.3) | 54 (8.0) | 53 (8.4) | 68 (11.0) |  |  |  | |
| Other Hispanic | | 165 (6.0) | 32 (6.9) | 39 (5.2) | 43 (4.9) | 51 (7.5) |  |  |  | |
| Non-Hispanic White | | 788 (61.8) | 198 (61.6) | 225 (67.3) | 192 (60.1) | 173 (56.6) |  |  |  | |
| Non-Hispanic Black | | 351 (13.4) | 118 (14.4) | 84 (13.3) | 94 (15.0) | 55 (10.5) |  |  |  | |
| Other Race | | 255 (10.0) | 52 (8.8) | 42 (6.2) | 63 (11.6) | 98 (14.4) |  |  |  | |
| Education, No. (Weighted %)^a^ | | |  |  |  |  | 23.67 |  | 0.467 | |
| Less than 9th grade | | 209 (9.5) | 43 (7.5) | 58 (13.0) | 56 (8.5) | 52 (8.3) |  |  |  | |
| 9-11 grade | | 503 (25.1) | 136 (25.0) | 135 (26.3) | 115 (24.5) | 117 (24.1) |  |  |  | |
| High school graduate or equivalent | | 423 (24.0) | 111 (22.6) | 99 (24.4) | 118 (25.7) | 95 (23.1) |  |  |  | |
| Some college or AA degree | | 304 (17.5) | 85 (21.9) | 80 (16.1) | 66 (15.4) | 73 (17.0) |  |  |  | |
| College graduate or above | | 338 (23.9) | 68 (23.0) | 72 (20.2) | 90 (25.9) | 108 (27.5) |  |  |  | |
| BMI, No. (Weighted %)^a^ | | |  |  |  |  | 37.41 |  | 0.008 | |
| Normal (< 25) | | 442 (23.5) | 122 (31.5) | 101 (35.0) | 105 (45.0) | 114 (42.2) |  |  |  | |
| Overweight (25-30) | | 662 (38.3) | 165 (38.4) | 180 (45.3) | 151 (31.8) | 166 (36.6) |  |  |  | |
| Obesity (> 30) | | 673 (38.2) | 156 (30.1) | 163 (19.7) | 189 (12.2) | 165 (21.2) |  |  |  | |
| Smoking status, No. (Weighted %)^a^ | | |  |  |  |  | 13.70 |  | 0.044 | |
| Yes | | 382 (18.9) | 125 (22.7) | 104 (21.2) | 86 (17.1) | 67 (13.7) |  |  |  | |
| No | | 1395 (81.1) | 318 (77.3) | 340 (78.8) | 359 (82.9) | 378 (86.3) |  |  |  | |
| Physical activity, No. (Weighted %)^a^ | | |  |  |  |  | 4.74 |  | 0.500 | |
| Yes | | 350 (20.8) | 96 (22.1) | 79 (19.6) | 102 (23.5) | 73 (17.9) |  |  |  | |
| No | | 1427 (79.2) | 347 (77.9) | 365 (80.4) | 343 (76.5) | 372 (82.1) |  |  |  | |
| Diabetes, No. (Weighted %)^a^ | | |  |  |  |  | 0.067 |  | 0.997 | |
| Yes | | 362 (16.9) | 98 (17.2) | 82 (16.8) | 91 (16.9) | 91 (16.5) |  |  |  | |
| No | | 1415 (83.1) | 345 (82.8) | 362 (83.2) | 354 (83.1) | 354 (83.5) |  |  |  | |
| Hypertension, No. (Weighted %)^a^ | | |  |  |  |  | 4.622 |  | 0.267 | |
| Yes | | 878 (45.0) | 220 (43.2) | 224 (44.2) | 226 (49.4) | 208 (43.2) |  |  |  | |
| No | | 899 (55.0) | 223 (56.8) | 220 (55.8) | 219 (50.6) | 237 (56.8) |  |  |  | |
| Alcohol Consumption, No. (Weighted %)^a^ | | | | | | | 3.43 |  | 0.725 | |
| ≥ 12 times per year | | 247 (13.8) | 61 (11.3) | 60 (14.4) | 68 (15.4) | 58 (13.9) |  |  |  | |
| < 12 times per year | | 1530 (86.2) | 382 (88.7) | 384 (85.6) | 377 (84.6) | 387 (86.1) |  |  |  | |
| Exposure to secondhand smoke, No. (Weighted %)^a^ | | | | | | | 2.44 |  | 0.685 | |
| Yes | | 452 (22.5) | 126 (23.8) | 119 (24.1) | 103 (20.8) | 104 (21.0) |  |  |  | |
| No | | 1325 (77.5) | 317 (76.2) | 325 (75.9) | 342 (79.2) | 341 (79.0) |  |  |  | |
| Sedentary behavior, No. (Weighted %)^a^ | | | | | | | 6.21 |  | 0.332 | |
| Yes | | 1025 (60.3) | 272 (63.5) | 256 (56.1) | 254 (60.2) | 243 (62.3) |  |  |  | |
| No | | 752 (39.7) | 171 (36.5) | 188 (43.9) | 191 (39.8) | 202 (37.7) |  |  |  | |
| Arthritis, No. (Weighted %)^a^ | | | | | | | 8.69 |  | 0.193 | |
| Yes | | 677 (39.4) | 169 (35.6) | 183 (43.7) | 174 (36.5) | 151 (41.3) |  |  |  | |
| No | | 1100 (60.6) | 274 (64.4) | 261 (56.3) | 271 (63.5) | 294 (58.7) |  |  |  | |
| Thyroid problems, No. (Weighted %)^a^ | | | | | | | 10.31 |  | 0.245 | |
| Yes | | 253 (16.4) | 51 (14.2) | 63 (18.4) | 61 (13.1) | 78 (20.1) |  |  |  | |
| No | | 1774 (83.6) | 392 (85.8) | 381 (81.6) | 384 (86.9) | 367 (79.9) |  |  |  | |
| Hypercholesterolemia, No. (Weighted %)^a^ | | | | | | | 6.24 |  | 0.448 | |
| Yes | | 763 (44.4) | 176 (40.0) | 180 (45.3) | 194 (44.0) | 213 (48.5) |  |  |  | |
| No | | 1014 (55.6) | 267 (60.0) | 264 (54.7) | 251 (56.0) | 232 (51.5) |  |  |  | |
| GFR, No. (Weighted %)^a^ | | | | | | | 16.67 |  | 0.263 | |
| < 60 mL/min/1.73 m^2^ | | 265 (13.2) | 87 (16.5) | 70 (13.6) | 60 (10.8) | 48 (11.8) |  |  |  | |
| 60-90 mL/min/1.73 m^2^ | | 810 (47.7) | 202 (46.6) | 214 (52.1) | 200 (46.5) | 194 (44.4) |  |  |  | |
| ≥ 90 mL/min/1.73 m^2^ | | 702 (39.1) | 154 (36.9) | 160 (34.3) | 185 (42.7) | 203 (43.8) |  |  |  | |
| Annual household income, No. (Weighted %)^a^ | | | | | | | 4.30 |  | 0.973 | |
| $0 to $19,999 | | 327 (11.2) | 88 (11.8) | 91 (11.6) | 80 (11.5) | 68 (9.8) |  |  |  | |
| $20,000, to $34,999 | | 332 (13.8) | 104 (14.3) | 75 (13.9) | 77 (13.4) | 76 (13.4) |  |  |  | |
| $35,000 to $74,999 | | 421 (24.3) | 96 (25.0) | 102 (21.6) | 108 (24.9) | 115 (26.5) |  |  |  | |
| $75,000 and Over | | 697 (50.7) | 155 (48.9) | 176 (52.9) | 180 (50.2) | 186 (50.3) |  |  |  | |

^a^ Numbers of participants are unweighted. All percentage estimates are weighted

| **Table S6 Multivariate logistic analysis of osteoporosis in male participants of NHANES** | | | | | | | | | | | | |
| --- | --- | --- | --- | --- | --- | --- | --- | --- | --- | --- | --- | --- |
| **Characteristic** | |  | **Model 1** |  |  | **Model 2** | | |  |  | **Model 3** | |
|  |  | **OR** | **95% CI** | ***P*-value** |  | **OR** | **95% CI** | ***P*-value** |  | **OR** | **95% CI** | ***P*-value** |
| Age (≥ 60years） | | 2.49 | 1.05-5.90 | 0.038 |  | 2.42 | 0.83-7.01 | 0.104 |  | 2.06 | 0.71-6.01 | 0.189 |
| Race | |  |  |  |  |  |  |  |  |  |  |  |
| Mexican | | 0.50 | 0.11-2.20 | 0.355 |  | 0.60 | 0.09-3.98 | 0.598 |  | 0.46 | 0.05-4.02 | 0.484 |
| Other Hispanic | | 3.60 | 0.68-19.07 | 0.132 |  | 3.19 | 0.67-15.25 | 0.146 |  | 3.66 | 0.83-16.08 | 0.086 |
| Non-Hispanic White | | 1.07 | 0.36-3.25 | 0.900 |  | 1.18 | 0.37-3.76 | 0.783 |  | 1.73 | 0.49-6.06 | 0.394 |
| Non-Hispanic Black | | 1.71 | 0.47-6.20 | 0.415 |  | 2.02 | 0.53-7.62 | 0.300 |  | 3.21 | 0.76-·3.65 | 0.114 |
| Education | |  |  |  |  |  |  |  |  |  |  |  |
| Less than 9th grade | |  |  |  |  | 0.92 | 0.25-3.41 | 0.906 |  | 1.09 | 0.30-3.92 | 0.894 |
| 9-11 grade | |  |  |  |  | 0.73 | 0.21-2.54 | 0.622 |  | 0.75 | 0.22-2.57 | 0.652 |
| High school graduate or equivalent | |  |  |  |  | 0.81 | 0.31-2.12 | 0.673 |  | 0.78 | 0.28-2.14 | 0.624 |
| Some college or AA degree | |  |  |  |  | 0.26 | 0.06-1.02 | 0.053 |  | 0.22 | 0.08-1.68 | 0.048 |
| BMI | |  |  |  |  |  |  |  |  |  |  |  |
| Overweight (25-30) | |  |  |  |  | 0.28 | 0.09-0.87 | 0.029 |  | 0.25 | 0.09-0.67 | 0.006 |
| Obesity (> 30) | |  |  |  |  | 0.23 | 0.08-0.66 | 0.006 |  | 0.23 | 0.09-0.57 | 0.002 |
| Smoking status | |  |  |  |  |  |  |  |  | 0.37 | 0.08-1.68 | 0.198 |
| Physical activity | |  |  |  |  |  |  |  |  | 0.67 | 0.24-1.82 | 0.427 |
| Diabetes | |  |  |  |  |  |  |  |  | 0.25 | 0.03-1.93 | 0.182 |
| Hypertension | |  |  |  |  |  |  |  |  | 1.00 | 0.38-2.61 | 0.999 |
| Alcohol Consumption | |  |  |  |  |  |  |  |  | 2.11 | 0.77-5.78 | 0.148 |
| Exposure to secondhand smoke | |  |  |  |  |  |  |  |  | 0.70 | 0.20-2.46 | 0.577 |
| Sedentary behavior | |  |  |  |  |  |  |  |  | 1.01 | 0.37-2.58 | 0.982 |
| Arthritis | |  |  |  |  | 1.14 | 0.47-2.75 | 0.771 |  | 1.07 | 0.45-2.58 | 0.874 |
| Thyroid problems | |  |  |  |  | 2.21 | 0.53-9.32 | 0.280 |  | 2.74 | 0.51-14.80 | 0.242 |
| Hypercholesterolemia | |  |  |  |  |  |  |  |  | 1.33 | 0.73-2.43 | 0.351 |
| GFR |  |  |  |  |  |  |  |  |  |  |  |  |
| < 60 mL/min/1.73 m^2^ | |  |  |  |  | 1.26 | 0.37-4.28 | 0.717 |  | 1.41 | 0.32-6.19 | 0.651 |
| 60-90 mL/min/1.73 m^2^ | |  |  |  |  | 0.64 | 0.25-1.64 | 0.350 |  | 0.65 | 0.26-1.67 | 0.375 |
| Annual household income | |  |  |  |  |  |  |  |  |  |  |  |
| $0 to $19,999 | |  |  |  |  | 2.04 | 0.35-11.88 | 0.429 |  | 2.23 | 0.41-12.16 | 0.352 |
| $20,000, to $34,999 | |  |  |  |  | 1.92 | 0.45-8.17 | 0.376 |  | 2.21 | 0.55-8.91 | 0.263 |
| $35,000 to $74,999 | |  |  |  |  | 2.66 | 0.67-10.57 | 0.163 |  | 2.89 | 0.63-13.30 | 0.173 |
| Pb |  |  |  |  |  |  |  |  |  |  |  |  |
| Q2 | | 2.01 | 0.43-9.36 | 0.375 |  | 1.98 | 0.46-8.58 | 0.364 |  | 2.02 | 0.45-9.14 | 0.361 |
| Q3 | | 1.79 | 0.41-7.81 | 0.439 |  | 1.73 | 0.36-8.26 | 0.494 |  | 1.64 | 0.35-7.73 | 0.535 |
| Q4 | | 3.05 | 0.64-14.67 | 0.164 |  | 2.96 | 0.63-13.82 | 0.168 |  | 3.35 | 0.79-14.25 | 0.102 |
| Cd |  |  |  |  |  |  |  |  |  |  |  |  |
| Q2 | | 4.65 | 0.80-27.20 | 0.088 |  | 4.49 | 0.74-27.33 | 0.104 |  | 4.78 | 0.94-24.26 | 0.059 |
| Q3 | | 1.80 | 0.23-13.96 | 0.578 |  | 1.54 | 0.22-11.00 | 0.665 |  | 1.62 | 0.27-9.75 | 0.598 |
| Q4 | | 8.65 | 0.93-80.33 | 0.058 |  | 4.16 | 0.60-28.84 | 0.149 |  | 7.39 | 0.99-55.44 | 0.052 |
| Hg |  |  |  |  |  |  |  |  |  |  |  |  |
| Q2 | | 0.59 | 0.17-2.01 | 0.401 |  | 0.65 | 0.18-2.28 | 0.497 |  | 0.76 | 0.20-2.81 | 0.678 |
| Q3 | | 0.53 | 0.23-1.21 | 0.132 |  | 0.50 | 0.20-1.21 | 0.123 |  | 0.46 | 0.15-1.42 | 0.178 |
| Q4 | | 0.24 | 0.06-0.92 | 0.037 |  | 0.28 | 0.08-0.98 | 0.047 |  | 0.19 | 0.04-0.90 | 0.037 |
| Se |  |  |  |  |  |  |  |  |  |  |  |  |
| Q2 | | 0.50 | 0.14-1.78 | 0.282 |  | 0.52 | 0.18-1.49 | 0.222 |  | 0.47 | 0.20-1.08 | 0.075 |
| Q3 | | 0.25 | 0.08-0.76 | 0.015 |  | 0.33 | 0.11-1.05 | 0.061 |  | 0.31 | 0.10-0.92 | 0.035 |
| Q4 | | 0.13 | 0.03-0.53 | 0.005 |  | 0.19 | 0.04-0.81 | 0.025 |  | 0.16 | 0.04-0.63 | 0.009 |
| Mn |  |  |  |  |  |  |  |  |  |  |  |  |
| Q2 | | 0.72 | 0.15-3.39 | 0.674 |  | 1.03 | 0.30-3.50 | 0.961 |  | 1.56 | 0.45-5.36 | 0.484 |
| Q3 | | 1.01 | 0.16-6.37 | 0.995 |  | 1.27 | 0.23-6.95 | 0.785 |  | 1.63 | 0.40-6.57 | 0.493 |
| Q4 | | 2.10 | 0.56-7.84 | 0.272 |  | 2.29 | 0.72-7.36 | 0.163 |  | 3.07 | 0.71-13.23 | 0.133 |

Model 1: Adjusted by Age, Gender, Race；

Model 2: Adjusted by Age, Gender, Race, Education, BMI, Arthritis, Thyroid problems, GFR, Annual household income;

Model 3: Adjusted by Age, Gender, Race, Education, Smoke, Diabetes, Hypertension, Physical activity, BMI, Alcohol consumption, Exposure to secondhand smoke, Sedentary behavior, Arthritis, Thyroid problems, Hypercholesterolemia, GFR, Annual household income;

All OR (95% CI) estimates are weighted.

| **Table S7 Multivariate logistic analysis of osteoporosis in female participants of NHANES** | | | | | | | | | | | | |
| --- | --- | --- | --- | --- | --- | --- | --- | --- | --- | --- | --- | --- |
| **Characteristic** | |  | **Model 1** |  |  | **Model 2** | | |  |  | **Model 3** | |
|  |  | **OR** | **95% CI** | ***P*-value** |  | **OR** | **95% CI** | ***P*-value** |  | **OR** | **95% CI** | ***P*-value** |
| Age (≥ 60years） | | 4.52 | 2.39-8.55 | < 0.001 |  | 3.90 | 1.91-7.97 | < 0.001 |  | 3.77 | 1.76-8.09 | 0.001 |
| Race | |  |  |  |  |  |  |  |  |  |  |  |
| Mexican | | 0.19 | 0.04-0.83 | 0.027 |  | 0.11 | 0.03-0.41 | 0.001 |  | 0.09 | 0.02-0.36 | 0.001 |
| Other Hispanic | | 0.58 | 0.16-2.08 | 0.403 |  | 0.44 | 0.13-1.57 | 0.209 |  | 0.40 | 0.10-1.61 | 0.199 |
| Non-Hispanic White | | 0.53 | 0.16-1.75 | 0.297 |  | 0.53 | 0.14-2.00 | 0.346 |  | 0.47 | 0.13-1.75 | 0.261 |
| Non-Hispanic Black | | 0.39 | 0.11-1.39 | 0.144 |  | 0.40 | 0.09-1.69 | 0.212 |  | 0.37 | 0.09-1.50 | 0.165 |
| Education | |  |  |  |  |  |  |  |  |  |  |  |
| Less than 9th grade | |  |  |  |  | 3.30 | 0.89-12.22 | 0.073 |  | 4.88 | 1.24-19.22 | 0.024 |
| 9-11 grade | |  |  |  |  | 2.09 | 0.66-6.58 | 0.210 |  | 2.75 | 0.80-9.49 | 0.110 |
| High school graduate or equivalent | |  |  |  |  | 1.93 | 0.59-6.32 | 0.279 |  | 2.11 | 0.57-7.80 | 0.262 |
| Some college or AA degree | |  |  |  |  | 2.14 | 0.75-6.16 | 0.157 |  | 2.17 | 0.73-6.43 | 0.162 |
| BMI | |  |  |  |  |  |  |  |  |  |  |  |
| Overweight (25-30) | |  |  |  |  | 0.45 | 0.21-0.97 | 0.042 |  | 0.45 | 0.22-0.90 | 0.025 |
| Obesity (> 30) | |  |  |  |  | 0.21 | 0.08-0.52 | 0.001 |  | 0.19 | 0.07-0.50 | 0.001 |
| Smoking status | |  |  |  |  |  |  |  |  | 0.45 | 0.14-1.49 | 0.193 |
| Physical activity | |  |  |  |  |  |  |  |  | 1.01 | 0.36-2.79 | 0.993 |
| Diabetes | |  |  |  |  |  |  |  |  | 0.97 | 0.38-2.45 | 0.944 |
| Hypertension | |  |  |  |  |  |  |  |  | 1.24 | 0.59-2.61 | 0.570 |
| Alcohol Consumption | |  |  |  |  |  |  |  |  | 0.40 | 0.20-0.82 | 0.013 |
| Exposure to secondhand smoke | |  |  |  |  |  |  |  |  | 1.24 | 0.44-4.50 | 0.684 |
| Sedentary behavior | |  |  |  |  |  |  |  |  | 1.25 | 0.58-2.67 | 0.569 |
| Arthritis | |  |  |  |  | 1.38 | 0.70-2.76 | 0.355 |  | 1.35 | 0.69-2.68 | 0.383 |
| Thyroid problems | |  |  |  |  | 0.88 | 0.42-1.83 | 0.730 |  | 0.92 | 0.43-1.98 | 0.827 |
| Hypercholesterolemia | |  |  |  |  |  |  |  |  | 0.86 | 0.42-1.75 | 0.669 |
| GFR |  |  |  |  |  |  |  |  |  |  |  |  |
| < 60 mL/min/1.73 m^2^ | |  |  |  |  | 1.98 | 0.84-4.64 | 0.117 |  | 1.86 | 0.79-4.40 | 0.157 |
| 60-90 mL/min/1.73 m^2^ | |  |  |  |  | 1.12 | 0.55-2.30 | 0.754 |  | 1.16 | 0.57-2.39 | 0.681 |
| Annual household income | |  |  |  |  |  |  |  |  |  |  |  |
| $0 to $19,999 | |  |  |  |  | 1.83 | 0.63-5.28 | 0.264 |  | 1.69 | 0.55-5.16 | 0.360 |
| $20,000, to $34,999 | |  |  |  |  | 2.79 | 0.92-8.42 | 0.069 |  | 2.42 | 0.77-7.59 | 0.130 |
| $35,000 to $74,999 | |  |  |  |  | 2.76 | 1.19-6.40 | 0.019 |  | 2.78 | 1.19-6.47 | 0.018 |
| Pb |  |  |  |  |  |  |  |  |  |  |  |  |
| Q2 | | 0.67 | 0.29-1.55 | 0.350 |  | 0.57 | 0.22-1.47 | 0.248 |  | 0.56 | 0.20-1.53 | 0.255 |
| Q3 | | 0.72 | 0.31-1.66 | 0.441 |  | 0.51 | 0.23-1.14 | 0.099 |  | 0.59 | 0.26-1.34 | 0.207 |
| Q4 | | 0.50 | 0.24-1.06 | 0.069 |  | 0.30 | 0.13-0.68 | 0.004 |  | 0.31 | 0.12-0.77 | 0.012 |
| Cd |  |  |  |  |  |  |  |  |  |  |  |  |
| Q2 | | 8.89 | 1.79-44.25 | 0.008 |  | 14.43 | 2.13-97.56 | 0.006 |  | 14.11 | 2.12-94.13 | 0.006 |
| Q3 | | 21.92 | 4.35-89.79 | < 0.001 |  | 30.43 | 5.99-154.56 | < 0.001 |  | 30.55 | 5.90-158.11 | < 0.001 |
| Q4 | | 18.16 | 3.05-108.27 | 0.002 |  | 20.49 | 2.75-152.80 | 0.003 |  | 27.00 | 3.34-218.29 | 0.002 |
| Hg |  |  |  |  |  |  |  |  |  |  |  |  |
| Q2 | | 1.14 | 0.31-2.28 | 0.723 |  | 1.01 | 0.46-2.22 | 0.985 |  | 0.95 | 0.43-2.10 | 0.778 |
| Q3 | | 1.56 | 0.65-3.76 | 0.319 |  | 1.92 | 0.80-4.60 | 0.144 |  | 2.02 | 0.84-4.85 | 0.115 |
| Q4 | | 0.84 | 0.31-2.28 | 0.738 |  | 0.96 | 0.37-2.45 | 0.927 |  | 0.86 | 0.30-2.45 | 0.907 |
| Se |  |  |  |  |  |  |  |  |  |  |  |  |
| Q2 | | 0.48 | 0.23-1.06 | 0.068 |  | 0.42 | 0.17-1.03 | 0.059 |  | 0.44 | 0.18-1.08 | 0.072 |
| Q3 | | 0.50 | 0.19-1.28 | 0.145 |  | 0.60 | 0.23-1.56 | 0.295 |  | 0.59 | 0.23-1.51 | 0.268 |
| Q4 | | 0.26 | 0.15-0.45 | < 0.001 |  | 0.25 | 0.14-0.46 | < 0.001 |  | 0.27 | 0.14-0.53 | < 0.001 |
| Mn |  |  |  |  |  |  |  |  |  |  |  |  |
| Q2 | | 0.52 | 0.20-1.33 | 0.173 |  | 0.64 | 0.27-1.53 | 0.313 |  | 0.65 | 0.28-1.53 | 0.323 |
| Q3 | | 0.40 | 0.20-0.81 | 0.010 |  | 0.52 | 0.24-1.12 | 0.094 |  | 0.48 | 0.22-1.06 | 0.069 |
| Q4 | | 0.57 | 0.21-1.54 | 0.268 |  | 0.90 | 0.33-2.44 | 0.836 |  | 0.84 | 0.34-2.06 | 0.695 |

Model 1: Adjusted by Age, Gender, Race；

Model 2: Adjusted by Age, Gender, Race, Education, BMI, Arthritis, Thyroid problems, GFR, Annual household income;

Model 3: Adjusted by Age, Gender, Race, Education, Smoke, Diabetes, Hypertension, Physical activity, BMI, Alcohol consumption, Exposure to secondhand smoke, Sedentary behavior, Arthritis, Thyroid problems, Hypercholesterolemia, GFR, Annual household income;

All OR (95% CI) estimates are weighted.

| **Table S8 Multivariate logistic analysis of osteoporosis in smoking participants of NHANES** | | | | | | | | | | | | |
| --- | --- | --- | --- | --- | --- | --- | --- | --- | --- | --- | --- | --- |
| **Characteristic** | |  | **Model 1** |  |  | **Model 2** | | |  |  | **Model 3** | |
|  |  | **OR** | **95% CI** | ***P*-value** |  | **OR** | **95% CI** | ***P*-value** |  | **OR** | **95% CI** | ***P*-value** |
| Age (≥ 60years） | | 4.09 | 2.05-8.19 | < 0.001 |  | 4.30 | 2.03-9.14 | < 0.001 |  | 5.82 | 1.97-17.20 | 0.002 |
| Male | | 0.37 | 0.13-1.08 | 0.068 |  | 0.29 | 0.10-0.88 | 0.028 |  | 0.26 | 0.08-0.82 | 0.021 |
| Race | |  |  |  |  |  |  |  |  |  |  |  |
| Mexican | | 0.15 | 0.03-0.90 | 0.037 |  | 0.07 | 0.01-1.22 | 0.069 |  | 0.07 | 0.01-1.50 | 0.090 |
| Other Hispanic | | 1.75 | 0.30-10.10 | 0.533 |  | 1.19 | 0.11-13.02 | 0.888 |  | 1.30 | 0.09-18.37 | 0.846 |
| Non-Hispanic White | | 0.94 | 0.20-4.48 | 0.940 |  | 0.69 | 0.13-3.51 | 0.652 |  | 0.67 | 0.12-3.83 | 0.650 |
| Non-Hispanic Black | | 0.56 | 0.10-3.32 | 0.524 |  | 0.24 | 0.03-1.91 | 0.176 |  | 0.29 | 0.03-2.72 | 0.275 |
| Education | |  |  |  |  |  |  |  |  |  |  |  |
| Less than 9th grade | |  |  |  |  | 20.58 | 0.94-452.09 | 0.055 |  | 37.31 | 1.39-999 | 0.031 |
| 9-11 grade | |  |  |  |  | 13.98 | 0.78-252.20 | 0.074 |  | 32.51 | 1.19-888.54 | 0.039 |
| High school graduate or equivalent | |  |  |  |  | 15.41 | 0.92-259.95 | 0.058 |  | 29.52 | 1.70-513.45 | 0.020 |
| Some college or AA degree | |  |  |  |  | 13.16 | 0.59-293.73 | 0.104 |  | 24.28 | 1.28-460.13 | 0.034 |
| BMI | |  |  |  |  |  |  |  |  |  |  |  |
| Overweight (25-30) | |  |  |  |  | 0.09 | 0.01-0.59 | 0.012 |  | 0.08 | 0.01-0.67 | 0.020 |
| Obesity (> 30) | |  |  |  |  | 1.01 | 0.34-3.05 | 0.980 |  | 1.12 | 0.37-3.39 | 0.845 |
| Physical activity | |  |  |  |  |  |  |  |  | 1.59 | 0.49-5.16 | 0.443 |
| Diabetes | |  |  |  |  |  |  |  |  | 0.68 | 0.13-3.51 | 0.643 |
| Hypertension | |  |  |  |  |  |  |  |  | 0.63 | 0.17-2.41 | 0.503 |
| Alcohol Consumption | |  |  |  |  |  |  |  |  | 0.36 | 0.06-2.30 | 0.278 |
| Exposure to secondhand smoke | |  |  |  |  |  |  |  |  | 0.37 | 0.11-1.22 | 0.101 |
| Sedentary behavior | |  |  |  |  |  |  |  |  | 1.90 | 0.66-5.45 | 0.236 |
| Arthritis | |  |  |  |  | 2.35 | 0.73-7.57 | 0.151 |  | 2.55 | 0.76-8.63 | 0.131 |
| Thyroid problems | |  |  |  |  | 0.20 | 0.05-0.81 | 0.025 |  | 0.18 | 0.03-1.08 | 0.061 |
| Hypercholesterolemia | |  |  |  |  |  |  |  |  | 0.77 | 0.21-2.77 | 0.689 |
| GFR |  |  |  |  |  |  |  |  |  |  |  |  |
| < 60 mL/min/1.73 m^2^ | |  |  |  |  | 1.24 | 0.32-4.83 | 0.756 |  | 1.28 | 0.23-6.70 | 0.778 |
| 60-90 mL/min/1.73 m^2^ | |  |  |  |  | 0.95 | 0.29-3.11 | 0.932 |  | 0.96 | 0.31-2.97 | 0.946 |
| Annual household income | |  |  |  |  |  |  |  |  |  |  |  |
| $0 to $19,999 | |  |  |  |  | 0.71 | 0.16-3.25 | 0.662 |  | 0.77 | 0.13-4.47 | 0.768 |
| $20,000, to $34,999 | |  |  |  |  | 1.55 | 0.29-8.33 | 0.609 |  | 1.74 | 0.33-9.32 | 0.516 |
| $35,000 to $74,999 | |  |  |  |  | 0.65 | 0.11-3.78 | 0.631 |  | 0.61 | 0.11-3.38 | 0.572 |
| Pb |  |  |  |  |  |  |  |  |  |  |  |  |
| Q2 | | 1.56 | 0.27-9.14 | 0.179 |  | 1.80 | 0.24-13.47 | 0.569 |  | 2.54 | 0.33-19.60 | 0.371 |
| Q3 | | 0.85 | 0.15-4.93 | 0.860 |  | 0.54 | 0.24-13.47 | 0.424 |  | 0.47 | 0.08-2.76 | 0.404 |
| Q4 | | 2.42 | 0.67-8.79 | 0.625 |  | 1.55 | 0.38-6.33 | 0.541 |  | 1.90 | 0.38-9,61 | 0.437 |
| Cd |  |  |  |  |  |  |  |  |  |  |  |  |
| Q2 | | 0.48 | 0.12-1.97 | 0.307 |  | 0.26 | 0.01-5.72 | 0.396 |  | 0.24 | 0.02-2.87 | 0.257 |
| Q3 | | - | - | < 0.001 |  | - | - | < 0.001 |  | - | - | < 0.001 |
| Q4 | | - | - | < 0.001 |  | - | - | < 0.001 |  | - | - | < 0.001 |
| Hg |  |  |  |  |  |  |  |  |  |  |  |  |
| Q2 | | 0.74 | 0.0.28-1.97 | 0.546 |  | 0.49 | 0.15-1.59 | 0.238 |  | 0.42 | 0.08-2.12 | 0.292 |
| Q3 | | 1.44 | 0.39-5.24 | 0.583 |  | 1.52 | 0.47-4.87 | 0.484 |  | 1.40 | 0.48-4.09 | 0.537 |
| Q4 | | 0.74 | 0.17-3.29 | 0.695 |  | 2.15 | 0.55-8.43 | 0.274 |  | 2.20 | 0.48-10.03 | 0.308 |
| Se |  |  |  |  |  |  |  |  |  |  |  |  |
| Q2 | | 0.99 | 0.39-2.48 | 0.989 |  | 0.74 | 0.31-1.78 | 0.499 |  | 0.53 | 0.21-1.33 | 0.178 |
| Q3 | | 0.39 | 0.08-1.94 | 0.259 |  | 0.29 | 0.06-1.43 | 0.129 |  | 0.18 | 0.03-1.03 | 0.054 |
| Q4 | | 0.01 | 0.001-0.10 | < 0.001 |  | 0.01 | < 0.01-0.07 | < 0.001 |  | 0.01 | 0.01-0.05 | < 0.001 |
| Mn |  |  |  |  |  |  |  |  |  |  |  |  |
| Q2 | | 0.41 | 0.12-1.39 | 0.151 |  | 0.36 | 0.09-1.47 | 0.155 |  | 0.43 | 0.11-1.79 | 0.248 |
| Q3 | | 0.19 | 0.05-0.79 | 0.022 |  | 0.20 | 0.04-0.99 | 0.048 |  | 0.17 | 0.03-0.89 | 0.036 |
| Q4 | | 1.37 | 0.27-7.02 | 0.704 |  | 2.56 | 0.59-11.14 | 0.212 |  | 2.41 | 0.61-9.48 | 0.209 |

Model 1: Adjusted by Age, Gender, Race；

Model 2: Adjusted by Age, Gender, Race, Education, BMI, Arthritis, Thyroid problems, GFR, Annual household income;

Model 3: Adjusted by Age, Gender, Race, Education, Smoke, Diabetes, Hypertension, Physical activity, BMI, Alcohol consumption, Exposure to secondhand smoke, Sedentary behavior, Arthritis, Thyroid problems, Hypercholesterolemia, GFR, Annual household income;

All OR (95% CI) estimates are weighted.

| **Table S9 Multivariate logistic analysis of osteoporosis in non-smoking participants of NHANES** | | | | | | | | | | | | |
| --- | --- | --- | --- | --- | --- | --- | --- | --- | --- | --- | --- | --- |
| **Characteristic** | |  | **Model 1** |  |  | **Model 2** | | |  |  | **Model 3** | |
|  |  | **OR** | **95% CI** | ***P*-value** |  | **OR** | **95% CI** | ***P*-value** |  | **OR** | **95% CI** | ***P*-value** |
| Age (≥ 60years） | | 3.47 | 1.72-7.03 | < 0.001 |  | 3.05 | 1.72-7.13 | < 0.001 |  | 3.73 | 1.84-7.56 | < 0.001 |
| Male | | 0.36 | 0.21-0.59 | < 0.001 |  | 0.37 | 0.22-0.61 | < 0.001 |  | 0.37 | 0.20-0.66 | 0.001 |
| Race | |  |  |  |  |  |  |  |  |  |  |  |
| Mexican | | 0.36 | 0.09-1.41 | 0.143 |  | 0.34 | 0.08-1.51 | 0.156 |  | 0.32 | 0.07-1.53 | 0.152 |
| Other Hispanic | | 1.73 | 0.38-7.94 | 0.482 |  | 1.56 | 0.34-7.21 | 0.570 |  | 1.36 | 0.29-6.30 | 0.694 |
| Non-Hispanic White | | 0.68 | 0.24-1.92 | 0.462 |  | 0.75 | 0.24-4.11 | 0.626 |  | 0.72 | 0.22-2.33 | 0.584 |
| Non-Hispanic Black | | 0.77 | 0.24-2.42 | 0.651 |  | 0.96 | 0.22-4.12 | 0.956 |  | 0.84 | 0.19-3.73 | 0.822 |
| Education | |  |  |  |  |  |  |  |  |  |  |  |
| Less than 9th grade | |  |  |  |  | 1.64 | 0.66-4.07 | 0.289 |  | 2.02 | 0.75-5.41 | 0.164 |
| 9-11 grade | |  |  |  |  | 1.28 | 0.47-3.48 | 0.626 |  | 1.48 | 0.48-4.54 | 0.495 |
| High school graduate or equivalent | |  |  |  |  | 1.19 | 0.47-3.01 | 0.713 |  | 1.23 | 0.43-3.55 | 0.705 |
| Some college or AA degree | |  |  |  |  | 1.19 | 0.42-3.39 | 0.738 |  | 1.27 | 0.45-3.57 | 0.655 |
| BMI | |  |  |  |  |  |  |  |  |  |  |  |
| Overweight (25-30) | |  |  |  |  | 0.53 | 0.26-1.08 | 0.079 |  | 0.52 | 0.28-0.97 | 0.041 |
| Obesity (> 30) | |  |  |  |  | 0.16 | 0.06-0.43 | < 0.001 |  | 0.15 | 0.05-0.42 | < 0.001 |
| Physical activity | |  |  |  |  |  |  |  |  | 0.34 | 0.11-1.03 | 0.057 |
| Diabetes | |  |  |  |  |  |  |  |  | 0.77 | 0.27-2.16 | 0.616 |
| Hypertension | |  |  |  |  |  |  |  |  | 1.31 | 0.72-2.38 | 0.381 |
| Alcohol Consumption | |  |  |  |  |  |  |  |  | 0.80 | 0.43-1.50 | 0.486 |
| Exposure to secondhand smoke | |  |  |  |  |  |  |  |  | 1.56 | 0.58-4.17 | 0.375 |
| Sedentary behavior | |  |  |  |  |  |  |  |  | 0.92 | 0.45-1.89 | 0.830 |
| Arthritis | |  |  |  |  | 1.26 | 0.68-2.34 | 0.470 |  | 1.22 | 0.68-2.21 | 0.503 |
| Thyroid problems | |  |  |  |  | 1.19 | 0.58-2.45 | 0.637 |  | 1.31 | 0.63-2.72 | 0.471 |
| Hypercholesterolemia | |  |  |  |  |  |  |  |  | 0.84 | 0.47-1.49 | 0.550 |
| GFR |  |  |  |  |  |  |  |  |  |  |  |  |
| < 60 mL/min/1.73 m^2^ | |  |  |  |  | 1.78 | 0.76-4.19 | 0.186 |  | 1.73 | 0.77-3.88 | 0.181 |
| 60-90 mL/min/1.73 m^2^ | |  |  |  |  | 0.77 | 0.36-1.64 | 0.501 |  | 0.79 | 0.39-1.60 | 0.504 |
| Annual household income | |  |  |  |  |  |  |  |  |  |  |  |
| $0 to $19,999 | |  |  |  |  | 2.16 | 0.94-4.95 | 0.069 |  | 1.89 | 0.82-4.37 | 0.137 |
| $20,000, to $34,999 | |  |  |  |  | 2.78 | 0.98-7.85 | 0.054 |  | 2.55 | 0.91-7.16 | 0.077 |
| $35,000 to $74,999 | |  |  |  |  | 4.02 | 1.85-8.75 | < 0.001 |  | 4.09 | 1.79-9.36 | 0.001 |
| Pb |  |  |  |  |  |  |  |  |  |  |  |  |
| Q2 | | 0.82 | 0.36-1.85 | 0.631 |  | 0.54 | 0.24-1.18 | 0.122 |  | 0.56 | 0.26-1.19 | 0.133 |
| Q3 | | 0.98 | 0.48-2.02 | 0.954 |  | 0.66 | 0.36-1.23 | 0.190 |  | 0.71 | 0.39-1.31 | 0.276 |
| Q4 | | 0.50 | 0.21-1.19 | 0.116 |  | 0.28 | 0.12-0.68 | 0.005 |  | 0.29 | 0.13-0.67 | 0.004 |
| Cd |  |  |  |  |  |  |  |  |  |  |  |  |
| Q2 | | 8.09 | 2.10-31.21 | 0.002 |  | 11.23 | 2.37-53.25 | 0.002 |  | 8.77 | 2.19-35.14 | 0.002 |
| Q3 | | 16.13 | 4.66-55.86 | < 0.001 |  | 21.15 | 4.75-94.29 | < 0.001 |  | 15.44 | 4.00-59.60 | < 0.001 |
| Q4 | | 19.02 | 2.71-133.42 | 0.003 |  | 20.11 | 2.33-173.78 | 0.006 |  | 13.98 | 1.90-102.74 | 0.010 |
| Hg |  |  |  |  |  |  |  |  |  |  |  |  |
| Q2 | | 0.92 | 0.45-1.89 | 0.823 |  | 0.88 | 0.40-1.95 | 0.752 |  | 0.86 | 0.38-1.92 | 0.709 |
| Q3 | | 1.04 | 0.52-2.12 | 0.905 |  | 0.98 | 0.47-2.07 | 0.966 |  | 0.95 | 0.47-1.91 | 0.878 |
| Q4 | | 0.53 | 0.20-1.42 | 0.205 |  | 0.50 | 0.20-1.27 | 0.146 |  | 0.46 | 0.18-1.15 | 0.098 |
| Se |  |  |  |  |  |  |  |  |  |  |  |  |
| Q2 | | 0.48 | 0.19-1.18 | 0.110 |  | 0.44 | 0.19-1.02 | 0.056 |  | 0.47 | 0.21-1.02 | 0.057 |
| Q3 | | 0.50 | 0.21-1.21 | 0.124 |  | 0.58 | 0.23-1.46 | 0.249 |  | 0.57 | 0.23-1.42 | 0.223 |
| Q4 | | 0.22 | 0.18-0.60 | < 0.001 |  | 0.33 | 0.17-0.65 | 0.001 |  | 0.35 | 0.18-0.69 | 0.002 |
| Mn |  |  |  |  |  |  |  |  |  |  |  |  |
| Q2 | | 0.53 | 0.19-1.45 | 0.214 |  | 0.65 | 0.26-1.62 | 0.357 |  | 0.61 | 0.25-1.50 | 0.279 |
| Q3 | | 0.52 | 0.21-1.28 | 0.154 |  | 0.67 | 0.24-1.86 | 0.440 |  | 0.60 | 0.22-1.69 | 0.336 |
| Q4 | | 0.54 | 0.19-1.50 | 0.234 |  | 0.81 | 0.31-2.16 | 0.678 |  | 0.78 | 0.30-2.06 | 0.618 |

Model 1: Adjusted by Age, Gender, Race；

Model 2: Adjusted by Age, Gender, Race, Education, BMI, Arthritis, Thyroid problems, GFR, Annual household income;

Model 3: Adjusted by Age, Gender, Race, Education, Smoke, Diabetes, Hypertension, Physical activity, BMI, Alcohol consumption, Exposure to secondhand smoke, Sedentary behavior, Arthritis, Thyroid problems, Hypercholesterolemia, GFR, Annual household income;

All OR (95% CI) estimates are weighted.
